# Robust Test Statistics Based on Restricted Minimum Rényi’s Pseudodistance Estimators

**DOI:** 10.3390/e24050616

**Published:** 2022-04-28

**Authors:** María Jaenada, Pedro Miranda, Leandro Pardo

**Affiliations:** Department of Statistics and Operation Research, Faculty of Mathematics, Interdisciplinary Mathematical Insititute, Complutense University of Madrid, Plaza Ciencias, 3, 28040 Madrid, Spain; mjaenada@ucm.es (M.J.); pmiranda@mat.ucm.es (P.M.)

**Keywords:** Rényi’s pseudodistance, minimum Rényi’s pseudodistance estimators, restricted minimum Rényi’s pseudodistance estimators, Rao-type tests, divergence-based tests

## Abstract

The Rao’s score, Wald and likelihood ratio tests are the most common procedures for testing hypotheses in parametric models. None of the three test statistics is uniformly superior to the other two in relation with the power function, and moreover, they are first-order equivalent and asymptotically optimal. Conversely, these three classical tests present serious robustness problems, as they are based on the maximum likelihood estimator, which is highly non-robust. To overcome this drawback, some test statistics have been introduced in the literature based on robust estimators, such as robust generalized Wald-type and Rao-type tests based on minimum divergence estimators. In this paper, restricted minimum Rényi’s pseudodistance estimators are defined, and their asymptotic distribution and influence function are derived. Further, robust Rao-type and divergence-based tests based on minimum Rényi’s pseudodistance and restricted minimum Rényi’s pseudodistance estimators are considered, and the asymptotic properties of the new families of tests statistics are obtained. Finally, the robustness of the proposed estimators and test statistics is empirically examined through a simulation study, and illustrative applications in real-life data are analyzed.

## 1. Introduction

Let ${\left(X,\beta_{X},P_{\theta }\right)}_{\theta \in \Theta }$ be the statistical space associated with the random variable X, where $\beta_{X}$ is the σ-field of Borel subsets A⊂X and ${\left\{P_{\theta }\right\}}_{\theta \in \Theta }$ is a family of probability distributions defined on the measurable space $\left(X,\beta_{X}\right),$ whit Θ an open subset of $R^{p}$ and p≥1. We assume that the probability measures $P_{\theta }$ are described by densities $f_{\theta }\left(x\right)=dP_{\theta }/d\mu \left(x\right),$ where μ is a σ-finite measure on $\left(X,\;\beta_{X}\right).$ Given a random sample $X_{1},\ldots ,X_{n},$ of the random variable *X* with density belonging to the parametric family $P_{\theta }$, the most popular estimator for the model parameter θ is the maximum likelihood estimator (MLE), which maximizes the likelihood function of the assumed model. The MLE has been widely studied in the literature for general statistical models, and it has been shown that, under certain regularity conditions, the sequence of MLEs of θ, ${\hat{\theta }}_{n},$ is asymptotically normal and it satisfies some desirable properties, such as consistency and asymptotic efficiency. That is, the MLE is the BAN (best asymptotically normal) estimator. However, in many popular statistical models, the MLE is markedly non-robust against deviations, even very small ones, from the parametric conditions.

To overcome the lack of robustness, minimum distance (or minimum divergence) estimators (MDEs) have been developed. MDEs have received growing attention in statistical inference because of their ability to conciliate efficiency and robustness. In parametric estimation, the role of divergence or distance measures is very intuitive: the estimates of the unknown parameters are obtained by minimizing a suitable divergence measure between the estimated from data and the assumed model distributions. There is a growing body of literature that recognizes the importance of MDEs in terms of robustness, without a significant loss of efficiency, with respect to the MLE. See, for instance, the works of Beran [1], Tamura and Boes [2], Simpson [3,4], Lindsay [5], Pardo [6], and Basu et al. [7] and the references therein.

Let *G* denote the unknown distribution function, with associated density g, underlying the data. The minimum divergence (distance) functional evaluated at *G*, T(G), is defined as

(1) $$d\left(g,f_{T(G)}\right)=\min_{\theta \in \Theta }d\left(g,f_{\theta }\right),$$

with $d(g,f_{\theta })$ being a distance or divergence measure between the densities *g* and $f_{\theta }.$ As the true distribution underlying the data is unknown, given a random sample, we could estimate the model parameter θ, substituting in the previous expression the true distribution *G* by its empirical estimation $G_{n}.$ Therefore, the MDE of θ is given by

(2) $${\hat{\theta }}_{n}=T\left(G_{n}\right),$$

When dealing with continuous models, it is convenient to consider families of divergence measures for which non-parametric estimators of the unknown density function are not needed. From this perspective, the density power divergence (DPD) family, leading to the minimum density power divergence estimators (MDPDEs) (see Basu et al. [7]), as well as the Rényi’s pseudodistance (RP), leading to the minimum Rényi’s pseudodistance estimators (MRPE) (see Broniatowski et al. [8]) between others, play an important role. The results presented in Broniatowski et al. [8] in the context of independent and identically distributed random variables were extended for the case of independent but not identically distributed random variables by Castilla et al. [9].

In many situations we have additional knowledge about the true parameter value, as it must satisfy certain constraints. Then, the restricted parameter space has the form

(3) $$\left\{\theta \in \Theta /\;g\left(\theta \right)=0_{r}\right\},$$

where $0_{r}$ denotes the null vector of dimension *r*, and $g:R^{p}\to R^{r}$ is a vector-valued function such that the p×r matrix

(4) $$G\left(\theta \right)=\frac{\partial g^{T}\left(\theta \right)}{\partial \theta }$$

exists and is continuous in θ, and rank $\left(G\left(\theta \right)\right)=r$. Here, superscript *T* represents the transpose of the matrix. In the following, the restricted parameter space given in (3) is denoted by $\Theta_{0},$ as in most situations, it will represent a composite null hypothesis.

The most popular estimator of θ under the non-linear constraint given in (3) is the restricted MLE (RMLE) that maximizes the likelihood function subject to the constraint $g\left(\theta \right)=0_{r}$ (see Silvey [10]). The RMLE encounters similar robustness problems to the MLE. To overcome such deficiency, the restricted MDPDEs (RMDPDEs) were introduced in Basu et al. [11] and their theoretical robustness properties were later studied in Ghosh [12].

The main purpose in this paper is extending the theory developed for the MRPE to the restricted parameter space setting, yielding to the restricted MRPE (RMPRE), where the parameter space has the form (3). The rest of the paper is as follows: In Section 2, MRPE is introduced. Section 3 presents RMPRE, and its asymptotic distribution as well as its influence function are obtained. In Section 4, two different test statistics for testing composite null hypothesis, based on the RMRPE, are developed, and explicit expressions of the statistics are presented for testing in normal populations. Section 5 presents a simulation study, where the robustness of the proposed estimators and test statistics is empirically shown. Section 6 deals with real-data situations. Finally, some conclusions are presented in Section 7.

## 2. Minimum Rényi Pseudodistance Estimators

In this section, we introduce the MRPE. We derive the estimating equations of the MRPE and recall its asymptotic distribution.

Let $X_{1},\ldots ,X_{n}$ be a random sample of size *n* from a population having true and unknown density function g, modeled by a parametric family of densities $f_{\theta }$ with $\theta \in \Theta \subset R^{p}.$ The RP between the densities $f_{\theta }$ and *g* is given, for τ>0, by

$$\begin{array}{cc} R_{\tau }\left(f_{\theta },g\right)= & \frac{1}{\tau +1}\log\left(\int f_{\theta }{\left(x\right)}^{\tau +1}dx\right)+\frac{1}{\tau \left(\tau +1\right)}\log\left(\int g{\left(x\right)}^{\tau +1}dx\right)\\ \; & -\frac{1}{\tau }\log\left(\int f_{\theta }{\left(x\right)}^{\tau }g\left(x\right)dx\right).. \end{array}$$

The RP can be defined for τ=0 taking continuous limits, yielding the expression

$$R_{0}\left(f_{\theta },g\right)=\lim_{\tau ↓0}R_{\tau }\left(f_{\theta },g\right)=\int g\left(x\right)\log\frac{g(x)}{f_{\theta }\left(x\right)}dx.$$

Then, the RP coincides with the Kullback–Leibler divergence (KL) between *g* and $f_{\theta }$, at τ=0 (see Pardo, 2006).

The RP was considered for the first time by Jones et al. [13]. Later Broniatowski et al. [8] established some useful properties of the divergence, such as the positivity of the RP for any two densities and for all values of the parameter τ,$R_{\tau }\left(f_{\theta },g\right)\geq 0$ and uniqueness of the minimum RP within a parametric family, that is, $R_{\tau }\left(f_{\theta },g\right)=0$ if and only if $f_{\theta }=g.$ The last property justifies the definition of the MRPEs as the minimizer of the RP between the assumed distribution and the empirical distribution of the data. It is interesting to note that the so-called RP by Broniatowski et al. [8] had been previously considered by Fujisawa and Eguchi [14] under the name of γ-cross entropy. In that paper, some appealing robustness properties of the estimators based on such entropy are shown.

Given a sample $X_{1},\ldots ,X_{n}$, from Broniatowski et al. [8] it can be seen that minimizing $R_{\tau }\left(f_{\theta },g\right)$ leads to the following definition.

**Definition** **1.** *Let ${\left(X,\;\beta_{X},\;f_{\theta }\right)}_{\theta \in \Theta \subset R^{p}}$ be a statistical space. The MRPE based on the random sample $X_{1},\ldots ,X_{n}$ for the unknown parameter **θ** is given, for τ>0, by*

(5) $${\hat{\theta }}_{\tau }\left(X_{1},\ldots ,X_{n}\right)=\arg\underset{\theta \in \Theta }{\sup}\sum_{i=1}^{n}\frac{f_{\theta }{\left(X_{i}\right)}^{\tau }}{C_{\tau }\left(\theta \right)},$$

*where*

$$C_{\tau }\left(\theta \right)={\left(\int f_{\theta }{\left(x\right)}^{\tau +1}dx\right)}^{\frac{\tau }{\tau +1}}.$$

Further, at τ=0,${\hat{\theta }}_{0}\left(X_{1},\ldots ,X_{n}\right)$ minimizes the KL divergence, and thus the MRPE coincides with the MLE for τ=0. Based on the previous definition (5), differentiating, we obtain that the estimating equations of the MRPE are given by

(6) $$\sum_{i=1}^{n}\Psi_{\tau }\left(x_{i};\theta \right)=0_{p},$$

with

(7) $$\begin{array}{cc} \Psi_{\tau }\left(x;\theta \right) & =f_{\theta }{\left(x\right)}^{\tau }\left(u_{\theta }\left(x\right)-c_{\tau }\left(\theta \right)\right),\\ u_{\theta }\left(x\right) & ={\left(u_{\theta_{1}}\left(x\right),\ldots ,u_{\theta_{p}}\left(x\right)\right)}^{T},\;u_{\theta_{i}}\left(x\right)=\frac{\partial }{\partial \theta_{i}}\log f_{\theta }\left(x\right),\\ \frac{\partial C_{\tau }\left(\theta \right)}{\partial \theta } & =C_{\tau }\left(\theta \right)c_{\tau }\left(\theta \right)\tau , \end{array}$$

being

(8) $$\begin{array}{cc} c_{\tau }\left(\theta \right) & =\frac{1}{\kappa_{\tau }\left(\theta \right)}\xi_{\tau }\left(\theta \right)={\left(c_{\tau ,1}\left(\theta \right),\ldots ,c_{\tau ,p}\left(\theta \right)\right)}^{T}, \end{array}$$

(9) $$\begin{array}{cc} \xi_{\tau }\left(\theta \right) & =\int f_{\theta }{\left(x\right)}^{\tau +1}u_{\theta }\left(x\right)dx, \end{array}$$

(10) $$\begin{array}{cc} \kappa_{\tau }\left(\theta \right) & =\int f_{\theta }{\left(x\right)}^{\tau +1}dx. \end{array}$$

The MRPE is an M-estimator and thus its asymptotic distribution and influence function (IF) can be obtained based on the asymptotic theory of the M-estimators. Broniatowski et al. [8] studied the asymptotic properties and robustness of the MRPEs. The next result recalls the asymptotic distribution of the MRPEs.

**Theorem** **1.** *Let $\theta_{0}$ be the true unknown value of θ. Then,*

(11) $$\sqrt{n}\left({\hat{\theta }}_{\tau }-\theta_{0}\right)\underset{n\to \infty }{\overset{L}{\to }}N\left(0_{p},V_{\tau }\left(\theta_{0}\right)\right)$$

*where*

(12) $$V_{\tau }\left(\theta \right)=S_{\tau }{\left(\theta \right)}^{-1}K_{\tau }\left(\theta \right)S_{\tau }{\left(\theta \right)}^{-1}$$

*with*

(13) $$\begin{array}{cc} S_{\tau }\left(\theta \right) & =-E\left[\frac{\partial \Psi_{\tau }{\left(X;\theta \right)}^{T}}{\partial \theta }\right], \end{array}$$

(14) $$\begin{array}{cc} K_{\tau }\left(\theta \right) & =E\left[\Psi_{\tau }\left(X;\theta \right)\Psi_{\tau }^{T}\left(X;\theta \right)\right]. \end{array}$$

Castilla et al. [15] introduced useful notation for the computation of $V_{\tau }\left(\theta \right).$

(15) $$\begin{array}{cc} S_{\tau }\left(\theta \right) & =J_{\tau }\left(\theta \right)-\frac{1}{\kappa_{\tau }\left(\theta \right)}\xi_{\tau }\left(\theta \right)\xi_{\tau }{\left(\theta \right)}^{T}, \end{array}$$

(16) $$\begin{array}{cc} K_{\tau }\left(\theta \right) & =J_{2\tau }\left(\theta \right)+\frac{1}{\kappa_{\tau }\left(\theta \right)}\left(\frac{\kappa_{2\tau }\left(\theta \right)}{\kappa_{\tau }\left(\theta \right)}\xi_{\tau }\left(\theta \right)\xi_{\tau }{\left(\theta \right)}^{T}-\xi_{\tau }\left(\theta \right)\xi_{2\tau }{\left(\theta \right)}^{T}-\xi_{2\tau }\left(\theta \right)\xi_{\tau }{\left(\theta \right)}^{T}\right), \end{array}$$

where

(17) $$J_{\tau }\left(\theta \right)=\int f_{\theta }{\left(x\right)}^{\tau +1}u_{\theta }\left(x\right)u_{\theta }{\left(x\right)}^{T}dx,$$

and $\kappa_{\tau }\left(\theta \right)$ and $\xi_{\tau }\left(\theta \right)$ are as in (9) and (10), respectively.

Toma and Leoni-Aubin [16] defined new robust and efficient measures based on the RP. Later, Toma et al. [17] considered the MRPE for general parametric models and developed a model selection criterion for regression models. Broniatowski et al. [8] applied the method to the multiple regression model (MRM) with random covariates. Subsequently, Castilla et al. [18] developed Wald-type tests based on MRPE for the MRM, and Castilla et al. [19] studied the MRPE for the MRM in the ultra-high dimensional set-up. Further, Jaenada and Pardo [20,21] considered the MRPE and Wald-type test statistics for generalized linear models (GLM). Despite Wald-type test statistics, there exist others relevant test statistics having an important role in the statistical literature: the likelihood-ratio and Rao (or score) tests, which are based on restricted estimators, usually the RMLE. Then, it makes sense to develop robust versions of these popular statistics based on the RMRPE.

## 3. The Restricted Minimum Rényi Pseudodistance Estimator: Asymptotic Distribution and Influence Function of RMRPE

In this section, we introduce the RMRPE and we derive its asymptotic distribution. Moreover, we study its robustness properties through its influence function (IF).

**Definition** **2.** *The RMRPE functional ${\tilde{T}}_{\tau }\left(G\right)$ evaluated at the distribution G is defined by*

$$R_{\tau }\left(g,f_{{\tilde{T}}_{\tau }\left(G\right)}\right)=\min_{\theta \in \Theta_{0}}R_{\tau }\left(g,f_{\theta }\right),$$

*given that such a minimum exists.*

*Accordingly, given random sample $X_{1},\ldots ,X_{n}$ from the distribution G, the RMRPE of **θ** is defined as*

$${\tilde{\theta }}_{\tau }=\arg\underset{\theta \in \Theta_{0}}{\sup}\sum_{i=1}^{n}\frac{f_{\theta }{\left(X_{i}\right)}^{\tau }}{C_{\tau }\left(\theta \right)}.$$

Next, the result states the asymptotic distribution of the RMRPE, ${\tilde{\theta }}_{\tau }={\tilde{T}}_{\tau }\left(G\right).$

**Theorem** **2.** *Suppose that the true distribution satisfies the conditions of the model and let us denote by $\theta_{0}\in \Theta_{0}$ the true parameter. Then, the RMRPE ${\tilde{\theta }}_{\tau }$ of **θ** obtained under the constraints $g\left(\theta \right)=0_{r}$ has distribution*

$$n^{1/2}\left({\tilde{\theta }}_{\tau }-\theta_{0}\right)\underset{n⟶\infty }{\overset{L}{⟶}}N\left(0_{p},\Sigma_{\tau }\left(\theta_{0}\right)\right)$$

*where*

$$\Sigma_{\tau }\left(\theta_{0}\right)=P_{\tau }^{*}\left(\theta_{0}\right)K_{\tau }\left(\theta_{0}\right)P_{\tau }^{*}{\left(\theta_{0}\right)}^{T},$$

(18) $$P_{\tau }^{*}\left(\theta_{0}\right)=S_{\tau }{\left(\theta_{0}\right)}^{-1}-Q_{\tau }\left(\theta_{0}\right)G{\left(\theta_{0}\right)}^{T}S_{\tau }{\left(\theta_{0}\right)}^{-1},$$

(19) $$Q_{\tau }\left(\theta_{0}\right)=S_{\tau }{\left(\theta_{0}\right)}^{-1}G\left(\theta_{0}\right){\left[G{\left(\theta_{0}\right)}^{T}S_{\tau }{\left(\theta_{0}\right)}^{-1}G\left(\theta_{0}\right)\right]}^{-1}.$$

*and $S_{\tau }\left(\theta_{0}\right)$ is defined in (13), evaluated at $\theta =\theta_{0}$.*

**Proof.**  See Appendix A. □

To analyze the robustness of an estimator, Hampel et al. [22] introduced the concept of the influence function (IF). Since then, the IF has been widely used in statistical literature to measure robustness in different statistical contexts. Intuitively, the IF describes the effect of an infinitesimal contamination of the model on the estimate. Then, IFs associated to locally robust (B-robust) estimators should be bounded. Let us now obtain the IF of RMRPE and analyze its boundedness to asses the robustness of the proposed estimators. We consider the contaminated model $g_{\varepsilon }\left(x\right)=\left(1-\varepsilon \right)f_{\theta }\left(x\right)+\varepsilon \Delta_{x},$ with $\Delta_{x}$ the indicator function in x, and we denote ${\tilde{\theta }}_{\tau ,\varepsilon }={\tilde{T}}_{\tau }\left(G_{\varepsilon }\right),$ being $G_{\varepsilon }$ the distribution function associated to $g_{\varepsilon }.$ By definition, ${\tilde{\theta }}_{\tau ,\varepsilon }$ is the minimizer of $R_{\tau }\left(g,f_{\theta }\right)$ subject to $g({\tilde{\theta }}_{\tau ,\varepsilon })=0.$ Following the same steps as in Theorem 5 in Broniatowski et al. [8], it can be seen that the influence function of ${\tilde{T}}_{\tau }$ in $f_{\theta }$ is given by

(20) $$IF\left(x,{\tilde{T}}_{\tau },\theta \right)=M_{\tau }{\left(\theta \right)}^{-1}\left[f_{\theta }{\left(x\right)}^{\tau }u_{\theta }\left(x\right)-c_{\tau }\left(\theta \right)f_{\theta }{\left(x\right)}^{\tau }\right],$$

where $c_{\tau }\left(\theta \right)$ was defined in (8) and

$$\begin{array}{cc} M_{\tau }\left(\theta \right) & =\frac{1}{\int f_{\theta }{\left(x\right)}^{\tau +1}dx}\left[\int f_{\theta }{\left(x\right)}^{\tau +1}dx\int f_{\theta }{\left(x\right)}^{\tau +1}u_{\theta }\left(x\right)u_{\theta }{\left(x\right)}^{T}dx\right.\\ \; & \left.-\left(\int f_{\theta }{\left(x\right)}^{\tau +1}u_{\theta }\left(x\right)dx\right){\left(\int f_{\theta }{\left(x\right)}^{\tau +1}u_{\theta }\left(x\right)dx\right)}^{T}\right], \end{array}$$

with the additional condition that $g({\tilde{\theta }}_{\tau ,\varepsilon })=0.$ Note that expression (20) corresponds to the IF of the unrestricted MRPE. Differentiating this last equation gives, at ε=0,

(21) $$G{\left(\theta \right)}^{T}IF\left(x,{\tilde{T}}_{\tau },\theta \right)=0.$$

Based on (20) and (21) we have

$$\left(\begin{array}{c} M_{\tau }\left(\theta \right)\\ G{\left(\theta \right)}^{T} \end{array}\right)IF\left(x,{\tilde{T}}_{\tau },\theta \right)=\left(\begin{array}{c} \left[f_{\theta }{\left(x\right)}^{\tau }u_{\theta }\left(x\right)-c_{\tau }\left(\theta \right)f_{\theta }{\left(x\right)}^{\tau }\right]\\ 0 \end{array}\right).$$

Therefore,

$$\left(\begin{array}{cc} M_{\tau }{\left(\theta \right)}^{T} & G\left(\theta \right) \end{array}\right)\left(\begin{array}{c} M_{\tau }\left(\theta \right)\\ G{\left(\theta \right)}^{T} \end{array}\right)IF\left(x,{\tilde{T}}_{\tau },\theta \right)=M_{\tau }{\left(\theta \right)}^{T}\left[f_{\theta }{\left(x\right)}^{\tau }u_{\theta }\left(x\right)-c_{\tau }\left(\theta \right)f_{\theta }{\left(x\right)}^{\tau }\right]$$

and

(22) $$IF\left(x,{\tilde{T}}_{\tau },\theta \right)={\left(M_{\tau }{\left(\theta \right)}^{T}M_{\tau }\left(\theta \right)+G\left(\theta \right)G{\left(\theta \right)}^{T}\right)}^{-1}M_{\tau }{\left(\theta \right)}^{T}\left[f_{\theta }{\left(x\right)}^{\tau }u_{\theta }\left(x\right)-c_{\tau }\left(\theta \right)f_{\theta }{\left(x\right)}^{\tau }\right].$$

Note that matrices $M_{\tau }\left(\theta \right)$ and $G\left(\theta \right)$ involved in the expression (22) are defined except for the model and tuning parameters θ and τ, and so the boundedness of the IF of the RMRPE depends, therefore, on the boundedness of the factor

$$\left[f_{\theta }{\left(x\right)}^{\tau }u_{\theta }\left(x\right)-c_{\tau }\left(\theta \right)f_{\theta }{\left(x\right)}^{\tau }\right].$$

Therefore, the boundedness of the IF of the RMRPE depends directly on the boundedness of IF of the MRPE, stated in (20). The IF of the MRPE has been widely studied for general statistical models, concluding that the MRPEs are robust for positive values of τ, and that such robustness increases with the tuning parameter. A whole discussion can be found in the work of Broniatowski et al. [8]. Hence, the same properties hold for RMRPEs.

## 4. Robust Test Statistics Based on RMRPEs

In this section, we develop two statistics based on the RMRPEs for testing composite null hypothesis, and their asymptotic distributions are obtained. Both procedures are particularized to standard deviation testing (with unknown mean) under normal populations, and explicit expressions of the test statistics are obtained.

### 4.1. Testing Based on Divergence Measures

In this section, we present the family of Rényi’s pseudodistance test statistics (RPTS) for testing the null hypothesis given in (3). This family of test statistics is given by

(23) $$T_{\gamma }\left({\hat{\theta }}_{\tau },{\tilde{\theta }}_{\tau }\right)=2nR_{\gamma }\left(f_{{\hat{\theta }}_{\tau }},f_{{\tilde{\theta }}_{\tau }}\right).$$

The RPTS, $T_{\gamma }\left({\hat{\theta }}_{\tau },{\tilde{\theta }}_{\tau }\right)$, can be understood as a measure between the best unrestricted estimator of the model parameter, and the best estimator satisfying the null hypothesis. Large values of the RPTS indicate that the model densities associated with the restricted and unrestricted estimators are far away one from the other, and so the null hypothesis is not supported by the observed data. Hence, we should reject $H_{0}$ for large enough $T_{\gamma }\left({\hat{\theta }}_{\tau },{\tilde{\theta }}_{\tau }\right)$. We can observe that the family of RPTS defined in (23) depends on two tuning parameters, τ and γ. The first is used for estimating the unknown parameters, while the second is applied to obtain the family of test statistics. The following theorem presents the asymptotic distribution of the family of RPTS defined in (23).

**Theorem** **3.** *The asymptotic distribution of $T_{\gamma }\left({\hat{\theta }}_{\tau },{\tilde{\theta }}_{\tau }\right)$ defined in (23) coincides, under the null hypothesis $H_{0}$ given in (3), with the distribution of the random variable*

$$\sum_{i=1}^{r}\lambda_{i}^{\tau ,\gamma }\left(\theta_{0}\right)Z_{i}^{2},$$

*where $Z_{1},\ldots ,Z_{r}$ are independent standard normal variables, $\lambda_{1}^{\tau ,\gamma }\left(\theta_{0}\right),\ldots ,\lambda_{r}^{\tau ,\gamma }\left(\theta_{0}\right)$ are the nonzero eigenvalues of $M_{\gamma ,\tau }\left(\theta_{0}\right)=A_{\gamma }\left(\theta_{0}\right)B_{\tau }\left(\theta_{0}\right)K_{\tau }\left(\theta_{0}\right)B_{\tau }\left(\theta_{0}\right)$ and k=r. The matrices $A_{\gamma }\left(\theta_{0}\right)$ and $B_{\tau }\left(\theta_{0}\right)$ are given by,*

(24) $$\begin{array}{ccc} A_{\gamma }\left(\theta_{0}\right) & = & \frac{S_{\gamma }\left(\theta_{0}\right)}{\kappa_{\tau }\left(\theta_{0}\right)}, \end{array}$$

(25) $$\begin{array}{ccc} B_{\tau }\left(\theta_{0}\right) & = & Q_{\tau }\left(\theta_{0}\right)G{\left(\theta_{0}\right)}^{T}S_{\tau }{\left(\theta_{0}\right)}^{-1}. \end{array}$$

**Proof.**  See Appendix A. □

#### Rényi’s Pseudodistance Test Statistics for Normal Populations

Under the $N(\mu ,\sigma^{2})$ model, consider the problem of testing

(26) $$H_{0}:\sigma =\sigma_{0}\;\mathrm{versus}\;H_{1}:\sigma \neq \sigma_{0}$$

where μ is an unknown nuisance parameter. In this case, the unrestricted and null parameter spaces are given by $\Theta =\{\left(\mu ,\sigma^{2}\right)\in R^{2}|\mu \in R,\sigma^{2}\in R^{+}\}$ and $\Theta_{0}=\left\{\left(\mu ,\sigma \right)\in R^{2}|\sigma =\sigma_{0},\mu \in R\right\}$, respectively. If we consider the function $g\left(\theta \right)=\sigma -\sigma_{0},$ with $\theta ={\left(\mu ,\sigma \right)}^{T}$, the null hypothesis $H_{0}$ can be written as

$$H_{0}:g\left(\theta \right)=0$$

and we are in the situation considered in (26). We can observe that in our case $G\left(\theta \right)={\left(0,1\right)}^{T}.$ Based on (6) and taking into account the fact that $f_{\theta }\left(x\right)$ is the normal density with mean μ and variance $\sigma^{2}$, the MRPE ${\hat{\theta }}_{\tau }={\left({\hat{\mu }}_{\tau },{\hat{\sigma }}_{\tau }\right)}^{T}$ of $\theta ={\left(\mu ,\sigma \right)}^{T}$ is the solution of the system of nonlinear equations

$$\left\{\begin{array}{c} \sum_{i=1}^{n}\left(X_{i}-\mu \right)\exp\left\{-\frac{\tau }{2}{\left(\frac{X_{i}-\mu }{\sigma }\right)}^{2}\right\}=0\\ \sum_{i=1}^{n}\left\{{\left(\frac{X_{i}-\mu }{\sigma }\right)}^{2}-\frac{1}{1+\tau }\right\}\exp\left\{-\frac{\tau }{2}{\left(\frac{X_{i}-\mu }{\sigma }\right)}^{2}\right\}=0 \end{array}\right.$$

while the RMRPE ${\tilde{\theta }}_{\beta }={\left({\tilde{\mu }}_{\tau },\sigma_{0}\right)}^{T},$ when $\sigma =\sigma_{0}$ is the solution of the nonlinear equation

$$\sum_{i=1}^{n}\left\{{\left(\frac{X_{i}-\mu }{\sigma_{0}}\right)}^{2}-\frac{1}{1+\tau }\right\}\exp\left\{-\frac{\tau }{2}{\left(\frac{X_{i}-\mu }{\sigma_{0}}\right)}^{2}\right\}=0.$$

After some algebra (see the Appendix A) we obtain that the RPTS for testing (26) under normal populations can be expressed as

(27) $$\begin{array}{ccc} T_{\gamma }\left({\hat{\theta }}_{\tau },{\tilde{\theta }}_{\tau }\right) & = & 2nR_{\gamma }\left(N\left({\hat{\mu }}_{\tau },{\hat{\sigma }}_{\tau }^{2}\right),N\left({\tilde{\mu }}_{\tau },\sigma_{0}\right)\right)\\ & = & \frac{2n}{\gamma \left(\gamma +1\right)}\log\left[\frac{1}{{\hat{\sigma }}_{\tau }\sigma_{0}^{\gamma }}{\left(\frac{\sqrt{{\hat{\sigma }}_{\tau }^{2}+\gamma \sigma_{0}^{2}}}{\sqrt{\gamma +1}}\right)}^{\gamma +1}\right]+n\frac{{\left({\hat{\mu }}_{\tau }-{\tilde{\mu }}_{\tau }\right)}^{2}}{\left(\gamma \sigma_{0}^{2}+{\hat{\sigma }}_{\tau }^{2}\right)} \end{array}$$

Based in (27), and taking into account that the eigenvalue of the matrix $A_{\gamma }\left(\theta \right)B_{\tau }\left(\theta \right)K_{\tau }\left(\theta \right)B_{\tau }\left(\theta \right)$ is given by (see Appendix A)

$$l_{\tau ,\gamma }\left(\sigma \right)=\frac{1}{2}\frac{{\left(\tau +1\right)}^{3}}{{\left(\gamma +1\right)}^{2}{\left(2\tau +1\right)}^{\frac{5}{2}}}\left(3\tau^{2}+4\tau +2\right),$$

we apply Theorem 3 such that

$$l_{\tau ,\gamma }{\left(\sigma_{0}\right)}^{-1}\left(\frac{2n}{\gamma \left(\gamma +1\right)}\log\left[\frac{1}{{\hat{\sigma }}_{\tau }\sigma_{0}^{\gamma }}{\left(\frac{\sqrt{{\hat{\sigma }}_{\tau }^{2}+\gamma \sigma_{0}^{2}}}{\sqrt{\gamma +1}}\right)}^{\gamma +1}\right]+n\frac{{\left({\hat{\mu }}_{\tau }-{\tilde{\mu }}_{\tau }\right)}^{2}}{\left(\gamma \sigma_{0}^{2}+{\hat{\sigma }}_{\tau }^{2}\right)}\right)\overset{L}{\underset{n\to \infty }{\to }}\chi_{1}^{2}.$$

Note that the RPTS is indexed by two tuning parameters, γ and τ, the first controlling the robustness of the pseudodistance and the second controlling the robustness on the estimation. For simplicity, we use γ=τ for the normal population application.

**Remark** **1.** *For τ=γ=0, the RPTS coincides with the asymptotic likelihood ratio test for testing (26). Indeed, for τ=0, we have that the MLE and RMLE are given, respectively, by*

$$\hat{\theta }=\left(\bar{X},{\hat{\sigma }}_{n}^{2}=\frac{1}{n}\sum_{i=1}^{n}{\left(X_{i}-\bar{X}\right)}^{2}\right)\;\mathrm{and}\;\;\tilde{\theta }=\left(\bar{X},\sigma_{0}^{2}\right).$$

*Now, the expression of the Kullback–Leibler divergence (the RP for γ=0) between two normal densities, $N(\mu_{1},\sigma_{1})$ and $N\left(\mu_{2},\sigma_{2}\right),$ is given by*

(28) $$\lim_{\gamma \to 0}R_{\gamma }\left(N\left(\mu_{1},\sigma_{1}\right),N\left(\mu_{2},\sigma_{2}\right)\right)=\frac{\sigma_{2}^{2}-\sigma_{1}^{2}}{2\sigma_{1}^{2}}+\ln\frac{\sigma_{1}}{\sigma_{2}}+\frac{1}{2}\frac{{\left(\mu_{1}-\mu_{2}\right)}^{2}}{\sigma_{1}^{2}}.$$

*and thus the RPTS for γ=τ=0 is*

$$T_{0}\left(\hat{\theta },\tilde{\theta }\right)=n\frac{\sigma_{0}^{2}}{{\hat{\sigma }}_{n}^{2}}-n+2n\ln\frac{{\hat{\sigma }}_{n}}{\sigma_{0}}.$$

*On the other hand, the likelihood ratio for testing (26) is given by*

$$\lambda \left(X_{1},\ldots ,X_{n}\right)={\left(\frac{{\hat{\sigma }}_{n}}{\sigma_{0}}\right)}^{n/2}e^{-n\frac{{\hat{\sigma }}_{n}^{2}}{2\sigma_{0}^{2}}}e^{n/2},$$

*and so, both expressions are related through*

$$-2\ln\lambda \left(X_{1},\ldots ,X_{n}\right)=T_{0}\left(\hat{\theta },\tilde{\theta }\right).$$

### 4.2. Rao’s-Type Tests Based on RMRPE

Rao test statistics are one of the most popular score test statistics for testing a simple and composite null hypothesis in general statistical models. For the simple null hypothesis testing, it requires no parameter estimation, but for composite ones, the classical Rao test is based on the likelihood score function associated with the restricted MLE (see Rao [23]). Basu et al. [24] generalized Rao’s procedure by using score functions associated with RMDPDEs, bringing in a considerable gain of robustness of the Rao-type test obtained. In this section, we develop Rao-type test statistics based on the score function associated to RMRPEs.

Let us consider the τ-score function associated to the RMRPE,

$$\psi_{\tau }\left(x;\theta \right)=f_{\theta }{\left(x\right)}^{\tau }\left(u_{\theta }\left(x\right)-c_{\tau }\left(\theta \right)\right),$$

so the estimating equations for the MRPE are given by

$$\sum_{i=1}^{n}\psi_{\tau }\left(x_{i};\theta \right)=0_{p}.$$

Then, the τ-score statistic can be defined as

$$\Psi_{\tau }\left(\theta \right)=\sum_{i=1}^{n}\psi_{\tau }\left(x_{i};\theta \right)={\left(\sum_{i=1}^{n}\psi_{\tau }^{1}\left(x_{i};\theta \right),\ldots ,\sum_{i=1}^{n}\psi_{\tau }^{k}\left(x_{i};\theta \right)\right)}^{T}.$$

However, taking expectations in the corresponding quantities, it is not difficult to show that

$$\begin{array}{cc} E\left[{\left(\frac{\tau }{C_{\tau }\left(\theta \right)}f_{\theta }{\left(X\right)}^{\tau }\left(u_{\theta }\left(X\right)-c_{\tau }\left(\theta \right)\right)\right)}_{\theta =\theta_{0}}\right] & =0_{p}\\ E\left[{\left(f_{\theta }{\left(X\right)}^{2\tau }\left(u_{\theta }\left(X\right)-c_{\tau }\left(\theta \right)\right){\left(u_{\theta }\left(X\right)-c_{\tau }\left(\theta \right)\right)}^{T}\right)}_{\theta =\theta_{0}}\right] & =K_{\tau }\left(\theta_{0}\right), \end{array}$$

where $K_{\tau }\left(\theta \right)$ is defined in (16), and so, by the central limit theorem, the τ-score statistic is asymptotically normal,

(29) $$\frac{1}{\sqrt{n}}\Psi_{\tau }\left(\theta \right)\underset{n\to \infty }{\overset{L}{\to }}N\left(0_{p},K_{\tau }\left(\theta \right)\right).$$

The previous convergence motivates the definition of the Rao-type test statistics.

#### 4.2.1. Rao-Type Test Statistics for Testing Simple Null Hypothesis

We first consider the simple null hypothesis test

(30) $$H_{0}:\theta =\theta_{0}\;\mathrm{vs}.\;H_{1}:\theta \neq \theta_{0}.$$

Then, the Rao-type test statistics $R_{\tau }\left(\theta_{0}\right)$ for testing (30) is defined as

$$R_{\tau }\left(\theta_{0}\right)=\frac{1}{n}\Psi_{\tau }{\left(\theta_{0}\right)}^{T}K_{\tau }{\left(\theta_{0}\right)}^{-1}\Psi_{\tau }\left(\theta_{0}\right).$$

Note that here the last test statistics depend on τ through the matrices $\Psi_{\tau }\left(\theta_{0}\right)$ and $K_{\tau }\left(\theta_{0}\right)$ involved in the definition, and again, the robustness of the statistics increases with τ. Moreover, the last matrix may have an explicit expression for certain statistical models, but otherwise it would have to be estimated from the sample.

Further, from (29), we have that, under the null hypothesis,

$$R_{\tau }\left(\theta_{0}\right)\underset{n\to \infty }{\overset{L}{\to }}\chi_{p}^{2}$$

with *p* being the dimension of the parameter space. Then, the null hypothesis is rejected if $R_{\tau }\left(\theta_{0}\right)>\chi_{p,\alpha }^{2}$, where $\chi_{p,\alpha }^{2}$ denotes the upper α-quantile of a chi-square distribution with *p* degrees of freedom.

#### 4.2.2. Rao-Type Test Statistics for Testing Composite Null Hypothesis

Next, let us consider composite null hypothesis of the form

(31) $$H_{0}:g\left(\theta \right)=0_{r}\;\mathrm{vs}.\;H_{1}:g\left(\theta \right)\neq 0_{r},$$

where the function $g:R^{p}\to R^{r}$ is a differentiable vector-valued function. Then, any vector θ satisfying the null hypothesis belongs to a restricted parameter space given in (3). The generalized Rao-type test statistic associated to the RMRPE with tuning parameter τ, ${\tilde{\theta }}_{\tau },$ for testing (31) is given by

(32) $$R_{\tau }\left({\tilde{\theta }}_{\tau }\right)=\frac{1}{n}\Psi_{\tau }{\left({\tilde{\theta }}_{\tau }\right)}^{T}Q_{\tau }\left({\tilde{\theta }}_{\tau }\right){\left[Q_{\tau }{\left({\tilde{\theta }}_{\tau }\right)}^{T}K_{\tau }\left({\tilde{\theta }}_{\tau }\right)Q_{\tau }\left({\tilde{\theta }}_{\tau }\right)\right]}^{-1}Q_{\tau }{\left({\tilde{\theta }}_{\tau }\right)}^{T}\Psi_{\tau }\left({\tilde{\theta }}_{\tau }\right).$$

Using similar arguments to Basu et al. [24], it is possible to show that, under general regularity conditions, the Rao-type test statistics $R_{\tau }\left({\tilde{\theta }}_{\tau }\right)$ have an asymptotic chi-square distribution with *r* degrees of freedom under the null hypothesis given in (31). Therefore, the rejection region of the test is given by

$$\{X_{1},\ldots ,X_{n}:R_{\tau }\left({\tilde{\theta }}_{\tau }\right)>\chi_{r,\alpha }^{2}\}.$$

Again, the tuning parameter τ controls the trade-off between efficiency and robustness of the test. Indeed, for τ=0, the generalized Rao type test statistic $R_{\tau =0}\left({\tilde{\theta }}_{0}\right)$ coincides with the classical Rao test for composite null hypothesis.

#### 4.2.3. Rao Test for Normal Populations

Consider the test defined in (26) for testing the standard deviation value of a normal population with unknown mean. The explicit expression of the main matrices involved in the definition (32) for such testing procedure and assumed parametric model is given by

$$\begin{array}{cc} \psi_{\tau }\left(X;\left(\mu ,\sigma \right)\right) & ={\left(\frac{X-\mu }{\sigma^{2}}\frac{1}{{\left(\sigma \sqrt{2\pi }\right)}^{\tau }}e^{-\frac{\tau }{2}{\left(\frac{X-\mu }{\sigma }\right)}^{2}},\left({\left(\frac{X-\mu }{\sigma }\right)}^{2}-\frac{1}{1+\tau }\right)\frac{1}{\sigma }\frac{1}{{\left(\sigma \sqrt{2\pi }\right)}^{\tau }}e^{-\frac{\tau }{2}{\left(\frac{X-\mu }{\sigma }\right)}^{2}}\right)}^{T},\\ K_{\tau }\left(\left(\mu ,\sigma \right)\right)\; & =\frac{1}{\sigma^{2}}\frac{1}{{\left(\sigma \sqrt{2\pi }\right)}^{2\tau }{\left(1+2\tau \right)}^{3/2}}\left(\begin{array}{cc} 1 & 0\\ 0 & \frac{3\tau^{2}+2+4\tau }{{\left(1+\tau \right)}^{2}\left(1+2\tau \right)} \end{array}\right),\\ Q_{\tau }\left(\left(\mu ,\sigma \right)\right)\; & =\left(\begin{array}{c} 0\\ 1 \end{array}\right). \end{array}$$

The step-by-step calculation of such values are detailed in the Appendix A. Then, the Rao-type test for composite null hypothesis of the form (31) is given by

$$R_{\tau }\left(\tilde{\mu }\right)=\frac{1}{n}\frac{{\left(1+2\tau \right)}^{3/2}{\left(1+\tau \right)}^{2}\left(1+2\tau \right)}{3\tau^{2}+4\tau +2}{\left[\sum_{i=1}^{n}\left({\left(\frac{x_{i}-\tilde{\mu }}{\sigma_{0}}\right)}^{2}-\frac{1}{\tau +1}\right)e^{-\frac{\tau }{2}{\left(\frac{x_{i}-\tilde{\mu }}{\sigma_{0}}\right)}^{2}}\right]}^{2}$$

where $\left({\tilde{\mu }}_{\tau },\sigma_{0}\right)$ denotes the RMRPE with tuning parameter τ. Note that, for τ=0, ${\tilde{\mu }}_{\tau =0}=\bar{X}.$ Then, the Rao-type test statistic based on RMRPE with τ=0 (the restricted MLE) coincides with the classical Rao test.

## 5. Simulation Study: Application to Normal Populations

In this section, we empirically analyze the performance of the proposed estimators under the normal parametric model and RPTS and Rao-type test statistics for the problem of testing (26) in terms of efficiency and robustness. We examine the accuracy of the RMRPEs, and we further examine the robustness properties of both families of estimators under different contamination scenarios. Further, we investigate the empirical level and power of the proposed test statistics under different sample sizes and contamination scenarios.

Let us consider a univariate normal model with true parameter value $\theta_{0}=(\mu \;=\;0,$σ=1), and the problem of testing

(33) $$H_{0}:\sigma =1\;\mathrm{vs}.\;H_{1}:\sigma \neq 1.$$

The restricted parameter space is then given by

$$\Theta_{0}=\left\{\left(\mu ,1\right):\mu \in R\right\}.$$

In order to evaluate the robustness properties of the estimators and test statistics, we introduce contamination in data by replacing a ε% of the observations by a contaminated sample, where ε denotes the contamination level. We generate five different scenarios of contamination:

- Pure data.
- Scenario 1: Slightly contaminated data. We replace a ε% of the samples by a contaminated sample from a normal distribution, $N(0,\sqrt{3}).$
- Scenario 2: Heavily contaminated data. We replace a ε% of the samples by a contaminated sample from a normal distribution, $N(0,\sqrt{5})$

Further, in order to evaluate the power of the test, we consider an alternative true parameter value $\theta_{1}=\left(0,0.7\right)$ which does not satisfy the null hypothesis (33) (or equivalently the restrictions of the parameter space). In this scenario, contaminated parameters are set $\theta_{1}=\left(0,1.2\right)$ for slightly and $\theta_{1}=\left(0,1.5\right)$ for heavily contamination.

**Figure 1:**
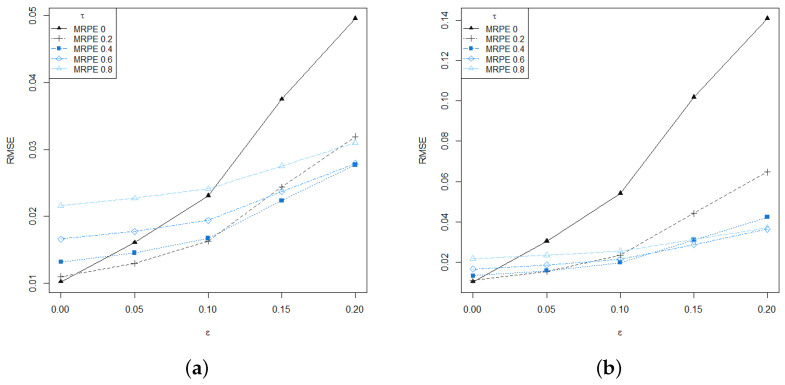
RMSE of the RMRPE under increasing contamination levels (slightly contaminated at left and heavily contaminated at right) for different values of the tuning parameter τ over R=10,000 replications. (**a**) Scenario 1, (**b**) Scenario 2.

Figure 1 shows the root mean square error (RMSE) of the RMRPE of the scale parameter σ, for different values of the tuning parameter τ=0,0.2,0.4,0.6 and τ=0.8 over R=10,000 replications. As expected, large values of the tuning parameter produce more robust estimators, which is particularly advantageous for the heavily contaminated scenario. Furthermore, even when introducing very low levels of contamination in data, ε=5%, the RMRPE with moderate value of the tuning parameter outperforms the classical MLE, without a significant loss of efficiency in the absence of contamination.

**Figure 2:**
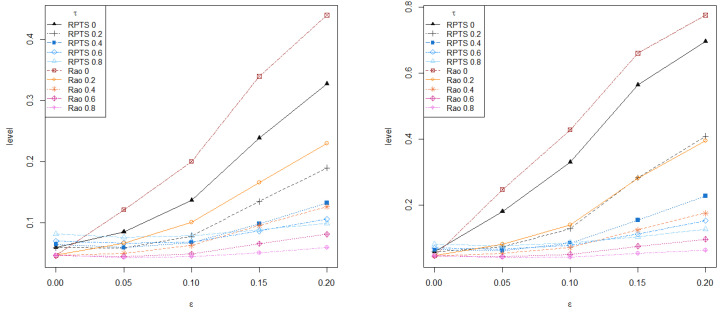

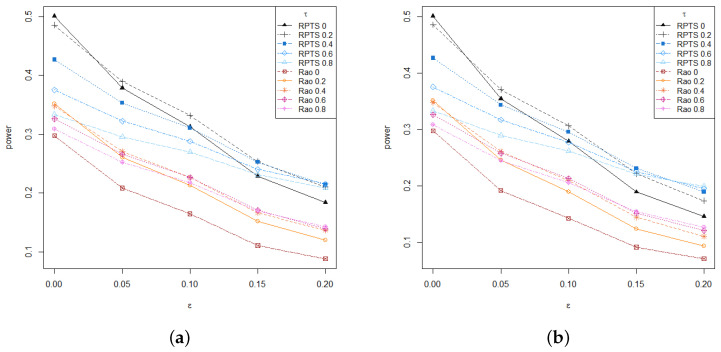
Empirical level and power under increasing contamination (slightly contaminated at left and heavily contaminated at right) over R=10,000 repetitions. (**a**) Scenario 1, (**b**) Scenario 2.

On the other hand, Figure 2 presents the empirical level and power of both RPTS and Rao-type test statistics based on RMRPEs for different values of the tuning parameter, τ=0,0.2,0.4,0.6,0.8, under increasing contamination levels. The empirical level and power are computed as the mean number of rejections over R=10,000 replications. The empirical level produced by the classical ratio and Rao-type tests rapidly increases and separates from levels obtained with any robust test. Regarding the empirical power, all robust tests with moderate and large values of the tuning parameter outperform the classical estimator within their family under contaminated scenarios, but Rao-type test statistics based on RMRPEs are more conservative than RPTSs, thus exhibiting lower levels and powers. Then, the proposed test statistics provides an appealing alternative to classical likelihood ratio and Rao tests, with a small loss of efficiency in favor of a clear gain in terms of robustness.

**Figure 3:**
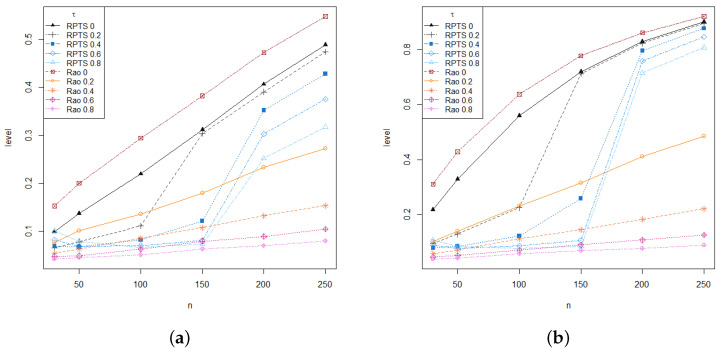
Empirical level under increasing sample sizes for 10% of contamination level (slightly contaminated at left and heavily contaminated at right) over R=10,000 repetitions. (**a**) Scenario 1, (**b**) Scenario 2.

On the other hand, the sample size could play a crucial role in the performance of the tests, even more accentuated when there exists data contamination. Figure 3 shows the sample size effect on the performance of the tests in terms of empirical level, under a 10% of contamination level in data. As discussed, Rao-type test statistics based on RMRPEs is more conservative and so tests based on RMRPEs with positive values of the tuning parameter produce lower empirical levels. Here, it outperforms the poor performance of the classical Rao-type test statistics with respect to any other. Moreover, when the sample size increases, the performance gap between non-robust and robust methods is widening.

Following the discussions in the preceding sections, larger values of the tuning parameter produce more robust but less efficient estimators. Therefore, the optimal value of τ should obtain the best trade-off between efficiency and robustness. Warwick and Jones [25] first introduced a useful data-based procedure for the choice of the tuning parameter for the MDPDE based on minimizing the asymptotic MSE of the estimator. However, this method depends on the choice of a pilot estimator, and Basak et al. [26] improved the method by removing the dependency on an initial estimator. The proposed algorithm was developed ad hoc for the MDPDE, but it can be easily adapted to the MRPE and RMRPE by simply substituting the expression of the variance of the MDPDE by the variance of the MRPPE or the RMRPE, respectively.

## 6. Real Data Application

Finally, we illustrate the outperformance of the proposed test statistics in two real data applications, where the gathered information contains some outlying observations. Both real dataset are modeled under the normal model, and hypothesis tests on the standard deviation of the population are performed.

### 6.1. Telephone-Fault Data

We consider the data on telephone line faults presented and analyzed by Welch [27] and Simpson [4]. The dataset consist of n=14 ordered differences between the inverse test rates and the inverse control rates in matched pairs of areas,

−988,−135,−78,3,59,83,93,110,189,197,204,229,289,310.

Basu et al. [24,28] modeled these differences as a normal random variable and pointed out that the first observation is a clear outlier, as its value is distant from the rest of the data. They tested simple and composite null hypotheses for the mean under the normal model, as well as a simple null hypothesis assuming a known mean. Here, we propose to test for the standard deviation of the normal distribution. Note that, computing the MLE of the sample with full and clean data (after removing the outlying observation), we obtain $\left(\hat{\mu },\hat{\sigma }\right)=\left(40.36,323.08\right),$ and $\left(\hat{\mu },\hat{\sigma }\right)=\left(119.46,134.82\right),$ respectively. Accordingly, the outlier clearly influences the model parameter estimates, playing a crucial role on the rejection of any null hypothesis. We consider the composite null hypothesis

(34) $$H_{0}:\sigma =135\;\mathrm{vs}.\;H_{1}:\sigma \neq 135,$$

where the value σ=135 has been chosen according to the estimation with clean data.

**Figure 4:**
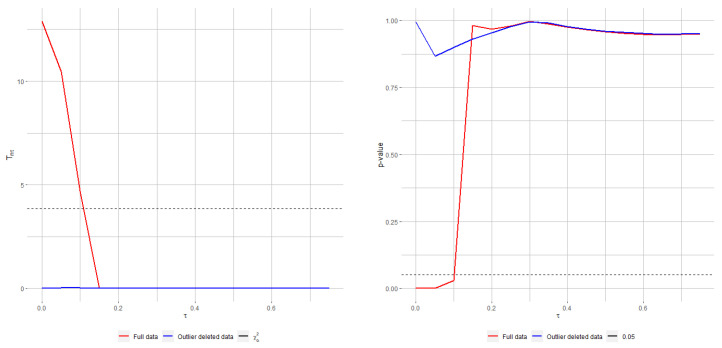

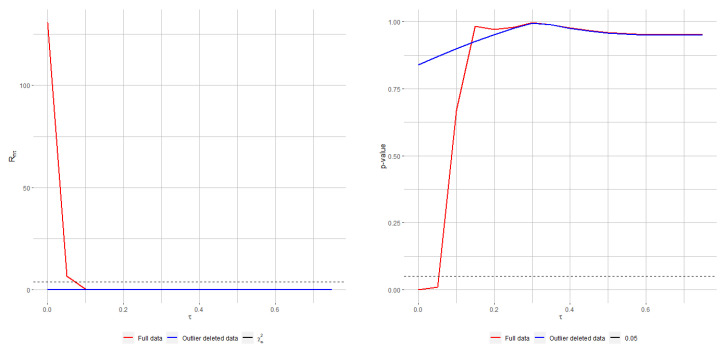
RPTS (**top**) and Rao-type test statistics (**bottom**), jointly with their associated *p*-valuess (right), for testing (34) with original and cleaned (after outliers removal) telephone-fault data.

Figure 4 presents the RPTS (top) and Rao (bottom) test statistics (left) and *p*-values (right) for the telephone data against increasing tuning parameters. While it is clearly seen that both classical tests fail to not reject the null hypothesis when fitting the model with the original data, the decision turns around sharply as the tuning parameter τ crosses and goes beyond 0.2 for the RPTS and 0.15 for Rao-type test statistics based on MRPEs. On the other hand, the decision of not rejecting is agreed by all statistics when fitting the model with clean data. This example illustrates the great applicability of the robust methods, which are not too affected by a such outlying observation, and the good performance of the proposed statistics under contaminated observations, which stay stable.

### 6.2. Darwin’s Plant Fertilization Data

Darwin [29] performed an experiment to determine whether self-fertilized plants and cross-fertilized plants have different growth rates. He sowed in pots pairs of *Zea mays* plants, one self-fertilized and the other cross-fertilized, and after a specific time period, the height of each plant was measured. A particular sample of n=15 pairs of plants led to the following paired differences (cross-fertilized minus self-fertilized).

−67,−48,6,8,14,16,23,24,28,29,41,49,56,60,75

A parametric approach to analyze the data as a random sample from a normal distribution with unknown mean and standard deviation was developed by Basu et al. [24]. Here, there is not any huge outlying observation, but the first two observations seem to be distant from the rest of the sample, influencing the model parameter estimates and test decisions. Indeed, the MLE, computing with original data, is $\left(\hat{\mu },\hat{\sigma }\right)=\left(20.93,37.74\right),$ while the MLE, when removing the two first observations, switches to $\left(\hat{\mu },\hat{\sigma }\right)=\left(33,21.54\right).$ Therefore, removing influential observations may alter the decision of a test. According to these results, we consider the testing problem

(35) $$H_{0}:\sigma =23\;\mathrm{vs}.\;H_{1}:\sigma \neq 23.$$

**Figure 5:**
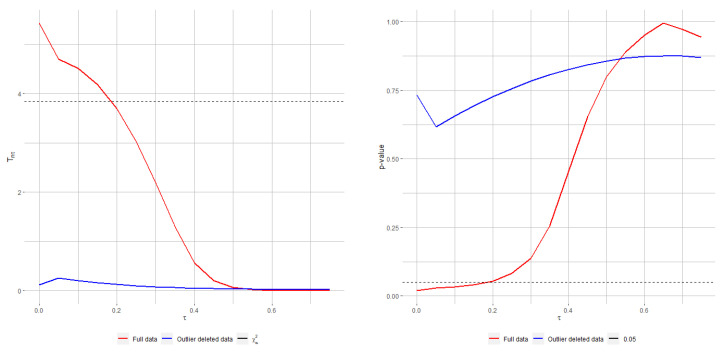

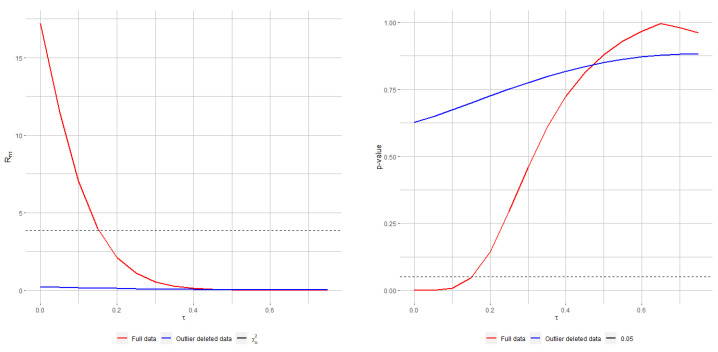
RPTS (**top**) and Rao-type test statistics (**bottom**), jointly with their associated *p*-values (right), for testing (35) with original and cleaned (after outliers removal) Darwing data.

Figure 5 shows the test statistics (left) and corresponding *p*-values (right) for the two families of statistics considered, the RPTS (top) and Rao-type test statistics (bottom) against the tuning parameter value τ. Again, test statistics based on RMRPE with large enough tuning parameters do not reject the null hypothesis, unlike tests based on low values of τ=0, including the RMLE. The disagreement departs when using the clean data, as all tests agree on not rejecting the null hypothesis.

## 7. Concluding Remarks

In this paper, we presented for the first time the family of RMRPEs. We derived their asymptotic distribution, and proved some suitable properties as consistency under the parameter restriction and robustness against data contamination. Further, based on these RMRPEs, we generalized two important families of statistics, namely RPTS and Rao-type tests, for testing a composite null hypothesis. Moreover, we obtained some explicit expressions of the RMPREs, RPTS and Rao-type test statistics for testing the variance under a normal population with an unknown mean. It was empirically shown that the proposed RPTS and Rao-type test statistics are robust, unlike classical tests based on the MLE, under normal populations. Indeed, the robustness of the tests is controlled by a tuning parameter τ, and so larger values of τ produce more robust estimators (although less efficient). Finally, some classical numerical examples illustrate the theoretical properties and applicability of the proposed methods.

## Data Availability

Not applicable.

## Abbreviations

The following abbreviations are used in this manuscript:

DPD	Density Power Divergence
IF	Influence Function
KL	Kullback–Leibler
LRM	Linear Regression Model
MLE	Maximum Likelihood Estimator
MDPDE	Minimum Density Power Divergence Estimator
MRPE	Minimum Rényi Pseudodistance Estimator
RMRPE	Restricted minimum Rényi Pseudodistance Estimator
RP	Rényi Pseudodistance
RPTS	Rényi Pseudodistance test statistic

## Appendix A

### Appendix A.1. Proof of Theorem 2

We denote

$$h_{n}\left(\theta \right)=\frac{1}{n}\sum_{i=1}^{n}\frac{f_{\theta }{\left(X_{i}\right)}^{\tau }}{C_{\tau }\left(\theta \right)}.$$

Differentiating both sides of the equality, we have

$$\frac{\partial h_{n}\left(\theta \right)}{\partial \theta }=\frac{\tau }{C_{\tau }\left(\theta \right)}\frac{1}{n}\sum_{i=1}^{n}f_{\theta }{\left(X_{i}\right)}^{\tau }\left(u_{\theta }\left(X_{i}\right)-c_{\tau }\left(\theta \right)\right).$$

Now we establish that

$${\left(\frac{\partial^{2}h_{n}\left(\theta \right)}{\partial \theta \partial \theta^{T}}\right)}_{\theta =\theta_{0}}\underset{n\to \infty }{\overset{P}{\to }}-\frac{\tau }{C_{\tau }\left(\theta \right)}S_{\tau }\left(\theta_{0}\right).$$

We have

$$\begin{array}{ccc} \frac{\partial^{2}h_{n}\left(\theta \right)}{\partial \theta \partial \theta^{T}} & = & \frac{1}{C_{\tau }{\left(\theta \right)}^{2}}\left\{\frac{1}{n}\sum_{i=1}^{n}\left[\left(\tau^{2}f_{\theta }{\left(X_{i}\right)}^{\tau }u_{\theta }\left(X_{i}\right)u_{\theta }{\left(X_{i}\right)}^{T}+\tau f_{\theta }{\left(X_{i}\right)}^{\tau }\frac{\partial u_{\theta }\left(X_{i}\right)}{\partial \theta^{T}}\right)C_{\tau }\left(\theta \right)\right.\right.\\ & & \left.\left.-\tau C_{\tau }\left(\theta \right)c_{\tau }\left(\theta \right)\tau f_{\theta }{\left(X_{i}\right)}^{\tau }u_{\theta }{\left(X_{i}\right)}^{T}\right]\right\}\\ & & -\frac{1}{C_{\tau }{\left(\theta \right)}^{2}}\left\{\frac{1}{n}\sum_{i=1}^{n}\left[\left(\tau \frac{\partial c_{\tau }\left(\theta \right)}{\partial \theta^{T}}f_{\theta }{\left(X_{i}\right)}^{\tau }+\tau^{2}f_{\theta }{\left(X_{i}\right)}^{\tau }c_{\tau }\left(\theta \right)u_{\theta }{\left(X_{i}\right)}^{T}\right)C_{\tau }\left(\theta \right)\right.\right.\\ & & \left.\left.-\tau C_{\tau }\left(\theta \right)c_{\tau }\left(\theta \right)\tau c_{\tau }{\left(\theta \right)}^{T}f_{\theta }{\left(X_{i}\right)}^{\tau }\right]\right\}\\ & = & \frac{1}{C_{\tau }\left(\theta \right)}\left\{\frac{1}{n}\sum_{i=1}^{n}\left[\tau^{2}f_{\theta }{\left(X_{i}\right)}^{\tau }u_{\theta }\left(X_{i}\right)u_{\theta }{\left(X_{i}\right)}^{T}+\tau f_{\theta }{\left(X_{i}\right)}^{\tau }\frac{\partial u_{\theta }\left(X_{i}\right)}{\partial \theta^{T}}\right.\right.\\ & & -\left.\left.\tau^{2}c_{\tau }{\left(\theta \right)}^{T}f_{\theta }{\left(X_{i}\right)}^{\tau }u_{\theta }{\left(X_{i}\right)}^{T}-\tau \frac{\partial c_{\tau }\left(\theta \right)}{\partial \theta^{T}}f_{\theta }{\left(X_{i}\right)}^{\tau }\right.\right.\\ & & \left.\left.-\tau^{2}c_{\tau }\left(\theta \right)f_{\theta }{\left(X_{i}\right)}^{\tau }u_{\theta }{\left(X_{i}\right)}^{T}-\tau^{2}c_{\tau }\left(\theta \right)c_{\tau }{\left(\theta \right)}^{T}f_{\theta }{\left(X_{i}\right)}^{\tau }\right]\right\}. \end{array}$$

As n→∞, we have

$${\left(\frac{\partial^{2}h_{n}\left(\theta \right)}{\partial \theta \partial \theta^{T}}\right)}_{\theta =\theta_{0}}\underset{n\to \infty }{\overset{P}{\to }}T\left(\theta_{0}\right)$$

with $T\left(\theta_{0}\right)$ being the matrix given by

$$\begin{array}{ccc} T\left(\theta_{0}\right) & = & \frac{1}{C_{\tau }\left(\theta \right)}\left\{\tau^{2}\int f_{\theta }{\left(x\right)}^{\tau +1}u_{\theta }\left(x\right)u_{\theta }{\left(x\right)}^{T}dx+\tau \int f_{\theta }{\left(x\right)}^{\tau +1}\frac{\partial u_{\theta }\left(x\right)}{\partial \theta^{T}}dx\right.\\ & & \left.-\tau^{2}c_{\tau }\left(\theta \right)\int f_{\theta }{\left(x\right)}^{\tau +1}u_{\theta }{\left(x\right)}^{T}dx-\tau \frac{\partial c_{\tau }\left(\theta \right)}{\partial \theta^{T}}\int f_{\theta }{\left(x\right)}^{\tau +1}dx\right.\\ & & \left.+\tau^{2}c_{\tau }\left(\theta \right)\int f_{\theta }{\left(x\right)}^{\tau +1}u_{\theta }{\left(x\right)}^{T}dx-\tau^{2}c_{\tau }\left(\theta \right)c_{\tau }{\left(\theta \right)}^{T}\int f_{\theta }{\left(x\right)}^{\tau +1}dx\right\}. \end{array}$$

From the above, after some algebra, we obtain

$$\begin{array}{ccc} T\left(\theta_{0}\right) & = & \frac{1}{C_{\tau }\left(\theta \right)}\left\{\tau^{2}\int f_{\theta }{\left(x\right)}^{\tau +1}u_{\theta }\left(x\right)u_{\theta }{\left(x\right)}^{T}dx+\tau \int f_{\theta }{\left(x\right)}^{\tau +1}\frac{\partial u_{\theta }\left(x\right)}{\partial \theta^{T}}dx\right.\\ & & \left.-\tau \frac{\partial c_{\tau }\left(\theta \right)}{\partial \theta^{T}}\int f_{\theta }{\left(x\right)}^{\tau +1}dx-\tau^{2}c_{\tau }{\left(\theta \right)}^{T}c_{\tau }\left(\theta \right)\int f_{\theta }{\left(x\right)}^{\tau +1}dx\right\}. \end{array}$$

On the other hand, it not difficult to establish that

$$\begin{array}{ccc} \frac{\partial c_{\tau }\left(\theta \right)}{\partial \theta^{T}} & = & \left(\tau +1\right)\frac{\int f_{\theta }{\left(x\right)}^{\tau +1}u_{\theta }\left(x\right)u_{\theta }{\left(x\right)}^{T}dx}{\int f_{\theta }{\left(x\right)}^{\tau +1}dx}+\frac{\int f_{\theta }{\left(x\right)}^{\tau +1}\frac{\partial u_{\theta }\left(x\right)}{\partial \theta^{T}}dx}{\int f_{\theta }{\left(x\right)}^{\tau +1}dx}\\ & & -\left(\tau +1\right)\frac{\int f_{\theta }{\left(x\right)}^{\tau +1}u_{\theta }\left(x\right)dx\int f_{\theta }{\left(x\right)}^{\tau +1}u_{\theta }{\left(x\right)}^{T}dx}{{\left(\int f_{\theta }{\left(x\right)}^{\tau +1}dx\right)}^{2}}. \end{array}$$

Therefore we have

$$\begin{array}{ccc} -\tau \frac{\partial c_{\tau }\left(\theta \right)}{\partial \theta^{T}}\int f_{\theta }{\left(x\right)}^{\tau +1}dx & = & -\tau \left(\tau +1\right)\int f_{\theta }{\left(x\right)}^{\tau +1}u_{\theta }\left(x\right)u_{\theta }{\left(x\right)}^{T}dx-\tau \int f_{\theta }{\left(x\right)}^{\tau +1}\frac{\partial u_{\theta }\left(x\right)}{\partial \theta^{T}}\\ & & +\tau \left(\tau +1\right)\frac{\int f_{\theta }{\left(x\right)}^{\tau +1}u_{\theta }\left(x\right)dx\int f_{\theta }{\left(x\right)}^{\tau +1}u_{\theta }{\left(x\right)}^{T}dx}{\int f_{\theta }{\left(x\right)}^{\tau +1}dx}. \end{array}$$

Finally,

$$\begin{array}{ccc} T\left(\theta_{0}\right) & = & \frac{1}{C_{\tau }\left(\theta \right)}\left\{\tau^{2}\int f_{\theta }{\left(x\right)}^{\tau +1}u_{\theta }\left(x\right)u_{\theta }{\left(x\right)}^{T}dx+\tau \int f_{\theta }{\left(x\right)}^{\tau +1}\frac{\partial u_{\theta }\left(x\right)}{\partial \theta^{T}}dx\right.\\ & & \left.-\tau \left(\tau +1\right)\int f_{\theta }{\left(x\right)}^{\tau +1}u_{\theta }\left(x\right)u_{\theta }{\left(x\right)}^{T}dx-\tau \int f_{\theta }{\left(x\right)}^{\tau +1}\frac{\partial u_{\theta }\left(x\right)}{\partial \theta^{T}}\right.\\ & & \left.+\tau \left(\tau +1\right)\frac{\int f_{\theta }{\left(x\right)}^{\tau +1}u_{\theta }\left(x\right)dx\int f_{\theta }{\left(x\right)}^{\tau +1}u_{\theta }{\left(x\right)}^{T}dx}{\int f_{\theta }{\left(x\right)}^{\tau +1}dx}-\tau^{2}c_{\tau }\left(\theta \right)c_{\tau }{\left(\theta \right)}^{T}\int f_{\theta }{\left(x\right)}^{\tau +1}dx\right\}\\ & = & \frac{1}{C_{\tau }\left(\theta \right)}\left\{-\tau \int f_{\theta }{\left(x\right)}^{\tau +1}u_{\theta }\left(x\right)u_{\theta }{\left(x\right)}^{T}dx+\tau \frac{\int f_{\theta }{\left(x\right)}^{\tau +1}u_{\theta }\left(x\right)dx\int f_{\theta }{\left(x\right)}^{\tau +1}u_{\theta }{\left(x\right)}^{T}dx}{\int f_{\theta }{\left(x\right)}^{\tau +1}dx}\right\}\\ & = & -\frac{\tau }{C_{\tau }\left(\theta \right)}S\left(\theta_{0}\right). \end{array}$$

On the other hand,

$$\sqrt{n}\frac{\partial h_{n}\left(\theta \right)}{\partial \theta }=\frac{\tau }{C_{\tau }\left(\theta \right)}\frac{1}{\sqrt{n}}\sum_{i=1}^{n}f_{\theta }{\left(X_{i}\right)}^{\tau }\left(u_{\theta }\left(X_{i}\right)-c_{\tau }\left(\theta \right)\right)\underset{n\to \infty }{\overset{L}{\to }}N\left(0_{p},{\left(\frac{\tau }{C_{\tau }\left(\theta \right)}\right)}^{2}K_{\tau }\left(\theta_{0}\right)\right),$$

as

$$E\left[{\left(\frac{\tau }{C_{\tau }\left(\theta \right)}f_{\theta }{\left(X\right)}^{\tau }\left(u_{\theta }\left(X\right)-c_{\tau }\left(\theta \right)\right)\right)}_{\theta =\theta_{0}}\right]=0_{p}$$

and

$$Cov\left[{\left(\frac{\tau }{C_{\tau }\left(\theta \right)}f_{\theta }{\left(X\right)}^{\tau }\left(u_{\theta }\left(X\right)-c_{\tau }\left(\theta \right)\right)\right)}_{\theta =\theta_{0}}\right]={\left(\frac{\tau }{C_{\tau }\left(\theta_{0}\right)}\right)}^{2}K_{\tau }\left(\theta_{0}\right)$$

Then, the RMRPE estimator of θ, ${\tilde{\theta }}_{\tau }$, must satisfy

(A1) $$\left\{\begin{array}{c} \frac{\partial }{\partial \theta }{\left.h_{n}\left(\theta \right)\right|}_{\theta ={\tilde{\theta }}_{\tau }}+G\left({\tilde{\theta }}_{\tau }\right)\lambda_{n}=0_{p},\\ g\left({\tilde{\theta }}_{\tau }\right)=0_{r}, \end{array}\right.$$

where $\lambda_{n}$ is a vector of Lagrangian multipliers. Now we consider $\theta_{n}=\theta_{0}+mn^{-1/2}$, with ||m||<k, for 0<k<∞. We have,

$$\frac{\partial }{\partial \theta }{\left.h_{n}\left(\theta \right)\right|}_{\theta =\theta_{n}}=\frac{\partial }{\partial \theta }{\left.h_{n}\left(\theta \right)\right|}_{\theta =\theta_{0}}+\frac{\partial }{\partial \theta^{T}}\frac{\partial }{\partial \theta }{\left.h_{n}\left(\theta \right)\right|}_{\theta =\theta_{0}}\left(\theta_{n}-\theta_{0}\right)+o(||\theta_{n}-\theta_{0}{\left|\right|}^{2})$$

and

(A2) $$n^{1/2}{\left.\frac{\partial }{\partial \theta }h_{n}\left(\theta \right)\right|}_{\theta =\theta_{n}}=n^{1/2}\frac{\partial }{\partial \theta }{\left.h_{n}\left(\theta \right)\right|}_{\theta =\theta_{0}}-\frac{\partial }{\partial \theta^{T}}\frac{\partial }{\partial \theta }{\left.h_{n}\left(\theta \right)\right|}_{\theta =\theta_{0}}n^{1/2}\left(\theta_{n}-\theta_{0}\right)+o(n^{1/2}\left|\right|\theta_{n}-\theta_{0}{\left|\right|}^{2}).$$

However,

$$o(n^{1/2}\left|\right|\theta_{n}-\theta_{0}{\left|\right|}^{2})=o(n^{1/2}{\left||m|\right|}^{2}/n)=o(n^{-1/2}{\left||m|\right|}^{2})=o\left(O_{p}\left(1\right)\right)=o_{p}\left(1\right).$$

Since

$$\lim_{n\to \infty }\frac{\partial }{\partial \theta^{T}}\frac{\partial }{\partial \theta }h_{n}{\left(\theta \right)|}_{\theta =\theta_{0}}=-\frac{\tau }{C_{\tau }\left(\theta \right)}S_{\tau }\left(\theta_{0}\right)$$

we obtain

(A3) $$n^{1/2}{\left.\frac{\partial }{\partial \theta }h_{n}\left(\theta \right)\right|}_{\theta =\theta_{n}}=n^{1/2}\frac{\partial }{\partial \theta }{\left.h_{n}\left(\theta \right)\right|}_{\theta =\theta_{0}}+\frac{\tau }{C_{\tau }\left(\theta \right)}S_{\tau }\left(\theta_{0}\right)n^{1/2}\left(\theta_{n}-\theta_{0}\right)+o_{p}\left(1\right).$$

Now, we know that

(A4) $$n^{1/2}g\left(\theta_{n}\right)=G{\left(\theta_{0}\right)}^{T}n^{1/2}\left(\theta_{n}-\theta_{0}\right)+o_{p}\left(1\right).$$

Further, the RMRPE ${\tilde{\theta }}_{\tau }$ must satisfy the conditions in (A1), and in view of (A3) and (A4) we have

(A5) $$n^{1/2}\frac{\partial }{\partial \theta }{\left.h_{n}\left(\theta \right)\right|}_{\theta =\theta_{0}}+\frac{\tau }{C_{\tau }\left(\theta \right)}S_{\tau }\left(\theta_{0}\right)n^{1/2}\left({\tilde{\theta }}_{\tau }-\theta_{0}\right)+G\left(\theta_{0}\right)n^{1/2}\lambda_{n}+o_{p}\left(1\right)=0_{p}.$$

From (A4) it follows that

(A6) $$G{\left(\theta_{0}\right)}^{T}n^{1/2}\left({\tilde{\theta }}_{\tau }-\theta_{0}\right)+o_{p}\left(1\right)=0_{r}.$$

Now we can express Equations (A5) and (A6) in matrix form as

$$\left(\begin{array}{cc} \frac{\tau }{C_{\tau }\left(\theta_{0}\right)}S_{\tau }\left(\theta_{0}\right) & G(\theta_{0})\\ G{\left(\theta_{0}\right)}^{T} & 0_{r\times r} \end{array}\right)\left(\begin{array}{c} n^{1/2}\left({\tilde{\theta }}_{\tau }-\theta_{0}\right)\\ n^{1/2}\lambda_{n} \end{array}\right)=\left(\begin{array}{c} -n^{1/2}\frac{\partial }{\partial \theta }{\left.h_{n}\left(\theta \right)\right|}_{\theta =\theta_{0}}\\ 0_{r} \end{array}\right)+o_{p}\left(1\right).$$

Therefore

$$\left(\begin{array}{c} n^{1/2}\left({\tilde{\theta }}_{\tau }-\theta_{0}\right)\\ n^{1/2}\lambda_{n} \end{array}\right)={\left(\begin{array}{cc} \frac{\tau }{C_{\tau }\left(\theta_{0}\right)}S_{\tau }\left(\theta_{0}\right)\; & G(\theta_{0})\\ G{\left(\theta_{0}\right)}^{T} & 0_{r\times r} \end{array}\right)}^{-1}\left(\begin{array}{c} -n^{1/2}\frac{\partial }{\partial \theta }{\left.h_{n}\left(\theta \right)\right|}_{\theta =\theta_{0}}\\ 0_{r} \end{array}\right)+o_{p}\left(1\right).$$

However,

$${\left(\begin{array}{cc} \frac{\tau }{C_{\tau }\left(\theta_{0}\right)}S_{\tau }\left(\theta_{0}\right)\; & G(\theta_{0})\\ G{\left(\theta_{0}\right)}^{T} & 0 \end{array}\right)}^{-1}=\left(\begin{array}{cc} {\left(\frac{\tau }{C_{\tau }\left(\theta_{0}\right)}\right)}^{-1}P_{\tau }^{*}\left(\theta_{0}\right) & Q_{\tau }\left(\theta_{0}\right)\\ Q_{\tau }{\left(\theta_{0}\right)}^{T} & R_{\tau }\left(\theta_{0}\right) \end{array}\right),$$

where $P_{\tau }^{*}\left(\theta_{0}\right)$ and $Q_{\tau }\left(\theta_{0}\right)$ are defined in (18) and (19), respectively. The matrix $R_{\tau }\left(\theta_{0}\right)$ is the quantity needed to make the right hand side of the above equation equal to the indicated inverse. Then,

(A7) $$n^{1/2}\left({\tilde{\theta }}_{\tau }-\theta_{0}\right)=-{\left(\frac{\tau }{C_{\tau }\left(\theta \right)}\right)}^{-1}P_{\tau }^{*}\left(\theta_{0}\right)n^{1/2}\frac{\partial }{\partial \theta }{\left.h_{n}\left(\theta \right)\right|}_{\theta =\theta_{0}}+o_{p}\left(1\right),$$

and we know

(A8) $$n^{1/2}\frac{\partial }{\partial \theta }{\left.h_{n}\left(\theta \right)\right|}_{\theta =\theta_{0}}\underset{n⟶\infty }{\overset{L}{⟶}}N\left(0,{\left(\frac{\tau }{C_{\tau }\left(\theta_{0}\right)}\right)}^{2}K_{\tau }\left(\theta_{0}\right)\right).$$

Now by (A7) and (A8), we have the desired result.

### Appendix A.2. Proof of Theorem 3

Consider the expression $R_{\gamma }\left(f_{\theta },f_{{\tilde{\theta }}_{\tau }}\right)$. A Taylor expansion for an arbitrary θ∈Θ, around ${\tilde{\theta }}_{\tau }$ leads to the relation

$$\begin{array}{cc} R_{\gamma }\left(f_{\theta },f_{{\tilde{\theta }}_{\tau }}\right) & =R_{\gamma }\left(f_{{\tilde{\theta }}_{\tau }},f_{{\tilde{\theta }}_{\tau }}\right)+{\left(\frac{\partial R_{\gamma }\left(f_{\theta },f_{{\tilde{\theta }}_{\tau }}\right)}{\partial \theta }\right)}_{\theta ={\tilde{\theta }}_{\tau }}\left(\theta -{\tilde{\theta }}_{\tau }\right)\\ \; & +\frac{1}{2}\left(\theta -{\tilde{\theta }}_{\tau }\right){\left(\frac{\partial^{2}R_{\gamma }\left(f_{\theta },f_{{\tilde{\theta }}_{\tau }}\right)}{\partial \theta \partial \theta^{T}}\right)}_{\theta ={\tilde{\theta }}_{\tau }}{\left(\theta -{\tilde{\theta }}_{\tau }\right)}^{T}+o\left({∥\theta -{\tilde{\theta }}_{\tau }∥}^{2}\right). \end{array}$$

It is clear that $R_{\gamma }\left(f_{{\tilde{\theta }}_{\tau }},f_{{\tilde{\theta }}_{\tau }}\right)=0$ and

$$\frac{\partial R_{\gamma }\left(f_{\theta },f_{{\tilde{\theta }}_{\tau }}\right)}{\partial \theta }=\frac{\partial L_{\gamma }^{1}\left(\theta \right)}{\partial \theta }-\frac{\partial L_{\gamma }^{2}\left(\theta \right)}{\partial \theta },$$

being

$$L_{\gamma }^{1}\left(\theta \right)=\frac{1}{\gamma +1}\log\left(\int f_{\theta }{\left(x\right)}^{\gamma +1}dx\right)$$

and

$$L_{\gamma }^{2}\left(\theta \right)=\frac{1}{\gamma }\log\left(\int f_{\theta }{\left(x\right)}^{\gamma }f_{{\tilde{\theta }}_{\tau }}\left(x\right)dx\right).$$

Then,

$$\frac{\partial L_{\gamma }^{1}\left(\theta \right)}{\partial \theta }=\frac{\int f_{\theta }{\left(x\right)}^{\gamma +1}u_{\theta }\left(x\right)dx}{\int f_{\theta }{\left(x\right)}^{\gamma +1}dx}\;\mathrm{and}\;\frac{\partial L_{\gamma }^{2}\left(\theta \right)}{\partial \theta }=\frac{\int f_{\theta }{\left(x\right)}^{\gamma }u_{\theta }\left(x\right)f_{{\tilde{\theta }}_{\tau }}\left(x\right)dx}{\int f_{\theta }{\left(x\right)}^{\gamma }f_{{\tilde{\theta }}_{\tau }}\left(x\right)dx}.$$

Therefore,

$${\left(\frac{\partial R_{\gamma }\left(f_{\theta },f_{{\tilde{\theta }}_{\tau }}\right)}{\partial \theta }\right)}_{\theta ={\tilde{\theta }}_{\tau }}=0.$$

Regarding the second derivatives, we have

$$\begin{array}{ccc} \frac{\partial^{2}L_{\gamma }^{1}\left(\theta \right)}{\partial \theta \partial \theta^{T}} & = & \left(\gamma +1\right)\frac{\int f_{\theta }{\left(x\right)}^{\gamma +1}u_{\theta }\left(x\right)u_{\theta }{\left(x\right)}^{T}dx}{\int f_{\theta }{\left(x\right)}^{\gamma +1}dx}+\frac{\int f_{\theta }{\left(x\right)}^{\gamma +1}\frac{\partial u_{\theta }\left(x\right)}{\partial \theta^{T}}}{\int f_{\theta }{\left(x\right)}^{\gamma +1}dx}\\ & & -\left(\gamma +1\right)\frac{\int f_{\theta }{\left(x\right)}^{\gamma +1}u_{\theta }\left(x\right)dx\int f_{\theta }{\left(x\right)}^{\gamma +1}u_{\theta }{\left(x\right)}^{T}dx}{{\left(\int f_{\theta }{\left(x\right)}^{\gamma +1}dx\right)}^{2}} \end{array}$$

and

$$\begin{array}{ccc} \frac{\partial^{2}L_{\gamma }^{2}\left(\theta \right)}{\partial \theta \partial \theta^{T}} & = & \gamma \frac{\int f_{\theta }{\left(x\right)}^{\gamma }u_{\theta }\left(x\right)u_{\theta }{\left(x\right)}^{T}f_{{\tilde{\theta }}_{\tau }}\left(x\right)dx}{\int f_{\theta }{\left(x\right)}^{\gamma }f_{{\tilde{\theta }}_{\tau }}\left(x\right)dx}+\frac{\int f_{\theta }{\left(x\right)}^{\gamma }\frac{\partial u_{\theta }\left(x\right)}{\partial \theta^{T}}f_{{\tilde{\theta }}_{\tau }}\left(x\right)dx}{\int f_{\theta }{\left(x\right)}^{\gamma }f_{{\tilde{\theta }}_{\tau }}\left(x\right)dx}\\ & & -\gamma \frac{\int f_{\theta }{\left(x\right)}^{\gamma }f_{{\tilde{\theta }}_{\tau }}\left(x\right)u_{\theta }\left(x\right)dx\int f_{\theta }{\left(x\right)}^{\gamma }f_{{\tilde{\theta }}_{\tau }}\left(x\right)u_{\theta }{\left(x\right)}^{T}dx}{{\left(\int f_{\theta }{\left(x\right)}^{\gamma }f_{{\tilde{\theta }}_{\tau }}\left(x\right)dx\right)}^{2}}. \end{array}$$

and so

$${\left(\frac{\partial^{2}R_{\gamma }\left(f_{\theta },f_{{\tilde{\theta }}_{\tau }}\right)}{\partial \theta \partial \theta^{T}}\right)}_{\theta ={\tilde{\theta }}_{\tau }}=\frac{S_{\gamma }\left({\tilde{\theta }}_{\tau }\right)}{\kappa_{\gamma }\left({\tilde{\theta }}_{\tau }\right)}$$

Therefore,

$$T_{\gamma }\left({\hat{\theta }}_{\tau },{\tilde{\theta }}_{\tau }\right)=2nR_{\gamma }\left(f_{\hat{\theta }},f_{{\tilde{\theta }}_{\tau }}\right)=n^{1/2}{\left({\hat{\theta }}_{\tau }-{\tilde{\theta }}_{\tau }\right)}^{T}\frac{S_{\gamma }\left({\tilde{\theta }}_{\tau }\right)}{\kappa_{\gamma }\left({\tilde{\theta }}_{\tau }\right)}n^{1/2}\left({\hat{\theta }}_{\tau }-{\tilde{\theta }}_{\tau }\right)+n\times o\left({∥{\hat{\theta }}_{\tau }-{\tilde{\theta }}_{\tau })∥}^{2}\right).$$

Under $\theta_{0}\in \Theta_{0}$,

$$\frac{S_{\gamma }\left({\tilde{\theta }}_{\tau }\right)}{\kappa_{\gamma }\left({\tilde{\theta }}_{\tau }\right)}\underset{n⟶\infty }{\overset{P}{⟶}}\frac{S_{\gamma }\left(\theta_{0}\right)}{\kappa_{\tau }\left(\theta_{0}\right)}.$$

Based on ${\hat{\theta }}_{\tau }$ and using by (A4) and (A5), we have that

$$n^{1/2}\frac{\partial }{\partial \theta }{\left.h_{n}\left(\theta \right)\right|}_{\theta =\theta_{0}}=-\frac{\tau }{C_{\tau }\left(\theta_{0}\right)}n^{1/2}S_{\tau }\left(\theta_{0}\right)\left({\hat{\theta }}_{\tau }-\theta_{0}\right)+o_{p}\left(1\right),$$

and using (A7), we obtain

$$\begin{array}{cc} n^{1/2}\left({\tilde{\theta }}_{\tau }-\theta_{0}\right) & =P_{\tau }^{*}\left(\theta_{0}\right)n^{1/2}S_{\tau }\left(\theta_{0}\right)\left({\hat{\theta }}_{\tau }-\theta_{0}\right)+o_{p}\left(1\right)\\ \; & =n^{1/2}\left({\hat{\theta }}_{\tau }-\theta_{0}\right)-Q_{\tau }\left(\theta_{0}\right)G{\left(\theta_{0}\right)}^{T}n^{1/2}\left({\hat{\theta }}_{\tau }-\theta_{0}\right)+o_{p}\left(1\right). \end{array}$$

Therefore,

(A9) $$n^{1/2}\left({\hat{\theta }}_{\tau }-{\tilde{\theta }}_{\tau }\right)=Q_{\tau }\left(\theta_{0}\right)G{\left(\theta_{0}\right)}^{T}n^{1/2}\left({\hat{\theta }}_{\tau }-\theta_{0}\right)+o_{p}\left(1\right).$$

On the other hand, we know that

$$n^{1/2}\left({\hat{\theta }}_{\tau }-\theta_{0}\right)\underset{n⟶\infty }{\overset{L}{⟶}}N\left(0,S_{\tau }{\left(\theta_{0}\right)}^{-1}K_{\tau }\left(\theta_{0}\right)S_{\tau }{\left(\theta_{0}\right)}^{-1}\right).$$

From Equations (19) and (25), we can establish that

$$\begin{array}{ccc} B_{\tau }\left(\theta_{0}\right) & = & S_{\tau }{\left(\theta_{0}\right)}^{-1}G\left(\theta_{0}\right){\left[G{\left(\theta_{0}\right)}^{T}S_{\tau }{\left(\theta_{0}\right)}^{-1}G\left(\theta_{0}\right)\right]}^{-1}G{\left(\theta_{0}\right)}^{T}S_{\tau }{\left(\theta_{0}\right)}^{-1}\\ & = & Q_{\tau }\left(\theta_{0}\right)G{\left(\theta_{0}\right)}^{-1}S_{\tau }{\left(\theta_{0}\right)}^{-1}. \end{array}$$

Therefore, it follows that

$$n^{1/2}\left({\hat{\theta }}_{\tau }-{\tilde{\theta }}_{\tau }\right)\underset{n⟶\infty }{\overset{L}{⟶}}N\left(0,B_{\tau }\left(\theta_{0}\right)K_{\tau }\left(\theta_{0}\right)B_{\tau }{\left(\theta_{0}\right)}^{T}\right).$$

Now, observe from the definition that $B_{\tau }\left(\theta_{0}\right)=B_{\tau }{\left(\theta_{0}\right)}^{T}.$

Then, the asymptotic distribution of the random variables

$$T_{\gamma }\left({\hat{\theta }}_{\tau },{\tilde{\theta }}_{\tau }\right)=2nR_{\gamma }\left(f_{{\hat{\theta }}_{\tau }},f_{{\tilde{\theta }}_{\tau }}\right)$$

and

$$n^{1/2}{\left({\hat{\theta }}_{\tau }-{\tilde{\theta }}_{\tau }\right)}^{T}\frac{S_{\gamma }\left(\theta_{0}\right)}{\kappa_{\gamma }\left(\theta_{0}\right)}n^{1/2}\left({\hat{\theta }}_{\tau }-{\tilde{\theta }}_{\tau }\right)$$

are the same, as we have established that

$$n\times o\left({∥{\hat{\theta }}_{\tau }-{\tilde{\theta }}_{\tau }∥}^{2}\right)=o_{p}\left(1\right).$$

Next, we apply Corollary 2.1 in Dik and Gunst [30], which states: “Let X be a *q*-variate normal random variable with mean vector 0 and variance-covariance matrix Σ. Let M be a real symmetric matrix of order *q*. Let k=rank(ΣMΣ), k≥1 and let $\lambda_{1},\ldots ,\lambda_{k},$ be the nonzero eigenvalues of MΣ. Then, the distribution of the quadratic form $X^{T}\mathrm{MX}$ coincides with the distribution of the random variable $\sum_{i=1}^{k}\lambda_{i}Z_{i}^{2},$ where $Z_{1},\ldots ,Z_{k}$ are independent, each having a standard normal variable”. In our case, the asymptotic distribution of $T_{\gamma }\left({\hat{\theta }}_{\tau },{\tilde{\theta }}_{\tau }\right)$ coincides with the distribution of the random variable $\sum_{i=1}^{k}\lambda_{i}^{\tau ,\gamma }\left(\theta_{0}\right)Z_{i}^{2}$ where $\lambda_{1}^{\tau ,\gamma }\left(\theta_{0}\right),\ldots ,\lambda_{k}^{\tau ,\gamma }\left(\theta_{0}\right)$, are the nonzero eigenvalues of $A_{\gamma }\left(\theta_{0}\right)B_{\tau }\left(\theta_{0}\right)K_{\tau }\left(\theta_{0}\right)B_{\tau }\left(\theta_{0}\right)$ and

(A10) $$k=\min\{r,rank\left(B_{\tau }\left(\theta_{0}\right)K_{\tau }\left(\theta_{0}\right)B_{\tau }\left(\theta_{0}\right)A_{\gamma }\left(\theta_{0}\right)B_{\tau }\left(\theta_{0}\right)K_{\tau }\left(\theta_{0}\right)B_{\tau }\left(\theta_{0}\right)\right)\}.$$

We now establish that k=r. The matrix,

$$N_{\tau }\left(\theta_{0}\right)=B_{\tau }\left(\theta_{0}\right)K_{\tau }\left(\theta_{0}\right)B_{\tau }\left(\theta_{0}\right)$$

is given by

$$\begin{array}{ccc} N_{\tau }\left(\theta_{0}\right) & = & S_{\tau }{\left(\theta_{0}\right)}^{-1}G\left(\theta_{0}\right){\left[G{\left(\theta_{0}\right)}^{T}S_{\tau }{\left(\theta_{0}\right)}^{-1}G\left(\theta_{0}\right)\right]}^{-1}G{\left(\theta_{0}\right)}^{T}S_{\tau }{\left(\theta_{0}\right)}^{-1}\\ & & K_{\tau }\left(\theta_{0}\right)S_{\tau }{\left(\theta_{0}\right)}^{-1}G\left(\theta_{0}\right){\left[G{\left(\theta_{0}\right)}^{T}S_{\tau }{\left(\theta_{0}\right)}^{-1}G\left(\theta_{0}\right)\right]}^{-1}G{\left(\theta_{0}\right)}^{T}S_{\tau }{\left(\theta_{0}\right)}^{-1}. \end{array}$$

Corollary 14.11.3 in Harville [31] (p. 259) establishes the following: “ For any m×n matrix A and any m×m symmetric positive definite matrix W, $rank(A^{T}W$A)=rank(A)". Based on this Corollary we have that $rank\left(N_{\tau }\left(\theta_{0}\right)\right)$ coincides with $rank\left(N_{\tau }\left(\theta_{0}\right)S_{\gamma }\left(\theta_{0}\right)N_{\tau }\left(\theta_{0}\right)\right).$

On the other hand, we know the following additional properties:

(a) rank(AB)=rank(A) if B is full rank (Corollary b.3.3 in Harville [31] (p. 83)).

(b) rank(AB)=rank(BA) if dimension of A coincides with dimension of $B^{T}.$

Matrix $K_{\tau }\left(\theta_{0}\right)$ should be “full rank”; in fact, if $K_{\tau }\left(\theta_{0}\right)$ were not full rank, the variance–covariance matrix of ${\hat{\theta }}_{\beta }$ and ${\tilde{\theta }}_{\beta }$ would not be full rank (there were redundant components in θ and this is not true).

Therefore, we have

$$\begin{array}{ccc} rank\left(N_{\tau }\left(\theta_{0}\right)\right) & = & {}_{\left(a\right)}rank\left(S_{\tau }{\left(\theta_{0}\right)}^{-\frac{1}{2}}G\left(\theta_{0}\right){\left[G{\left(\theta_{0}\right)}^{T}S_{\tau }{\left(\theta_{0}\right)}^{-1}G\left(\theta_{0}\right)\right]}^{-1}\right.\\ & & \left.G{\left(\theta_{0}\right)}^{T}S_{\tau }{\left(\theta_{0}\right)}^{-1}K_{\tau }\left(\theta_{0}\right)S_{\tau }{\left(\theta_{0}\right)}^{-1}G\left(\theta_{0}\right)\right.\\ & & \left.{\left[G{\left(\theta_{0}\right)}^{-1}S_{\tau }{\left(\theta_{0}\right)}^{-1}G\left(\theta_{0}\right)\right]}^{-1}G{\left(\theta_{0}\right)}^{T}S_{\tau }{\left(\theta_{0}\right)}^{-\frac{1}{2}}\right)\\ & = & {}_{\left(b\right)}rank\left(G{\left(\theta_{0}\right)}^{T}S_{\tau }{\left(\theta_{0}\right)}^{-1}K_{\tau }\left(\theta_{0}\right)S_{\tau }{\left(\theta_{0}\right)}^{-1}G\left(\theta_{0}\right){\left[G{\left(\theta_{0}\right)}^{T}S_{\tau }{\left(\theta_{0}\right)}^{-1}G\left(\theta_{0}\right)\right]}^{-1}\right)\\ & = & {}_{\left(a\right)}ran\left(G{\left(\theta_{0}\right)}^{T}S_{\tau }{\left(\theta_{0}\right)}^{-1}K_{\tau }\left(\theta_{0}\right)S_{\tau }{\left(\theta_{0}\right)}^{-1}G\left(\theta_{0}\right)\right)\\ & = & {}_{Corollary14.11.3}rank\left(S_{\tau }{\left(\theta_{0}\right)}^{-1}G\left(\theta_{0}\right)\right)\\ & = & {}_{\left(a\right)}rank\left(G\left(\theta_{0}\right)\right)=r. \end{array}$$

### Appendix A.3. Rényi’s Pseudodistance between Normal Populations

Here, we compute the expression of the RP between densities belonging to the normal model with parameters $\left(\mu_{1},\sigma_{1}\right)$ and $(\mu_{2},\sigma_{2}),$ respectively. The RP between $N(\mu_{1},\sigma_{1})$ and $N\left(\mu_{2},\sigma_{2}\right)$ is given by

$$\begin{array}{ccc} R_{\gamma }\left(N\left(\mu_{1},\sigma_{1}\right),N\left(\mu_{2},\sigma_{2}\right)\right) & = & \frac{1}{\gamma +1}\log\int N{\left(\mu_{1},\sigma_{1}\right)}^{\gamma +1}dx\\ & & +\frac{1}{\gamma \left(\gamma +1\right)}\log\int N{\left(\mu_{2},\sigma_{2}\right)}^{\gamma +1}dx-\frac{1}{\gamma }\log\int N{\left(\mu_{1},\sigma_{1}\right)}^{\gamma }N\left(\mu_{2},\sigma_{2}\right)dx\\ & = & \frac{1}{\gamma +1}\log L_{1}+\frac{1}{\gamma \left(\gamma +1\right)}\log L_{2}-\frac{1}{\gamma }\log L_{3}. \end{array}$$

We first compute

$$\int N{\left(\mu ,\sigma \right)}^{\beta }dx$$

for the seek of simplicity in later calculations.

$$\begin{array}{ccc} \int N{\left(\mu ,\sigma \right)}^{\beta }dx & = & \int {\left(\frac{1}{\sigma \sqrt{2\pi }}e^{-\frac{1}{2}{\left(\frac{x-\mu }{\sigma }\right)}^{2}}\right)}^{\beta }dx\\ & = & \frac{1}{\sigma^{\beta -1}{\left(\sqrt{2\pi }\right)}^{\beta -1}}\frac{1}{\sqrt{\beta }}\int \frac{1}{\frac{\sigma }{\sqrt{\beta }}\sqrt{2\pi }}e^{-\frac{1}{2}{\left(\frac{x-\mu }{\frac{\sigma }{\sqrt{\beta }}}\right)}^{2}}dx\\ & = & \frac{1}{\sigma^{\beta -1}{\left(\sqrt{2\pi }\right)}^{\beta -1}}\frac{1}{\sqrt{\beta }}. \end{array}$$

Therefore,

$$L_{1}=\frac{1}{\sigma_{1}^{\gamma }{\left(\sqrt{2\pi }\right)}^{\gamma }}\frac{1}{\sqrt{\gamma +1}}\;\mathrm{and}\;L_{2}=\frac{1}{\sigma_{2}^{\gamma }{\left(\sqrt{2\pi }\right)}^{\gamma }}\frac{1}{\sqrt{\gamma +1}}.$$

In relation with $L_{3}$ we have,

$$\begin{array}{ccc} L_{3} & = & \int N{\left(\mu_{1},\sigma_{1}\right)}^{\gamma }N\left(\mu_{2},\sigma_{2}\right)dx\\ & = & \int \frac{1}{\sigma_{1}^{\gamma }{\left(\sqrt{2\pi }\right)}^{\gamma }}e^{-\frac{1}{2}{\left(\frac{x-\mu_{1}}{\frac{\sigma_{1}}{\sqrt{\gamma }}}\right)}^{2}}\frac{1}{\sigma_{2}\sqrt{2\pi }}e^{-\frac{1}{2}{\left(\frac{x-\mu_{2}}{\sigma_{2}}\right)}^{2}}\\ & = & \frac{1}{\sigma_{1}^{\gamma }{\left(\sqrt{2\pi }\right)}^{\gamma }}\frac{1}{\sigma_{2}\sqrt{2\pi }}\times \\ & & \times \int \exp\left\{-\frac{1}{2}\left[x^{2}\left(\frac{1}{{\left(\frac{\sigma_{1}}{\sqrt{\gamma }}\right)}^{2}}+\frac{1}{\sigma_{2}^{2}}\right)-2x\left(\frac{\mu_{1}}{{\left(\frac{\sigma_{1}}{\sqrt{\gamma }}\right)}^{2}}+\frac{\mu_{2}}{\sigma_{2}^{2}}\right)+\frac{\mu_{1}^{2}}{{\left(\frac{\sigma_{1}}{\sqrt{\gamma }}\right)}^{2}}+\frac{\mu_{2}^{2}}{\sigma_{2}^{2}}\right]\right\}dx\\ & = & \frac{1}{\sigma_{1}^{\gamma }{\left(\sqrt{2\pi }\right)}^{\gamma }}\frac{1}{\sigma_{2}\sqrt{2\pi }}\exp\left\{-\frac{1}{2}\left[\frac{\mu_{1}^{2}}{{\left(\frac{\sigma_{1}}{\sqrt{\gamma }}\right)}^{2}}+\frac{\mu_{2}^{2}}{\sigma_{2}^{2}}\right]\right\}\times \\ & & \times \int \exp\left\{-\frac{1}{2}\left[x^{2}\left(\frac{1}{{\left(\frac{\sigma_{1}}{\sqrt{\gamma }}\right)}^{2}}+\frac{1}{\sigma_{2}^{2}}\right)-2x\left(\frac{\mu_{1}}{{\left(\frac{\sigma_{1}}{\sqrt{\gamma }}\right)}^{2}}+\frac{\mu_{2}}{\sigma_{2}^{2}}\right)\right]\right\}dx\\ & = & \frac{1}{\sigma_{1}^{\gamma }{\left(\sqrt{2\pi }\right)}^{\gamma }}\frac{1}{\sigma_{2}\sqrt{2\pi }}\exp\left\{-\frac{1}{2}\left[\frac{\mu_{1}^{2}}{{\left(\frac{\sigma_{1}}{\sqrt{\gamma }}\right)}^{2}}+\frac{\mu_{2}^{2}}{\sigma_{2}^{2}}\right]\right\}\exp\left\{\frac{1}{2}\frac{A^{2}}{B^{2}}\right\}B\sqrt{2\pi }\times \\ & & \times \int \frac{1}{\sqrt{2\pi }B}\exp\left\{-\frac{1}{2}{\left(\frac{x-A}{B}\right)}^{2}\right\}dx\\ & = & \frac{1}{\sigma_{1}^{\gamma }{\left(\sqrt{2\pi }\right)}^{\gamma }}\frac{1}{\sigma_{2}\sqrt{2\pi }}\exp\left\{-\frac{1}{2}\left[\frac{\mu_{1}^{2}}{{\left(\frac{\sigma_{1}}{\sqrt{\gamma }}\right)}^{2}}+\frac{\mu_{2}^{2}}{\sigma_{2}^{2}}\right]\right\}\exp\left\{\frac{1}{2}\frac{A^{2}}{B^{2}}\right\}B\sqrt{2\pi }. \end{array}$$

Now it is necessary to obtain *A* and B. However, for this, we have,

$$\left\{\begin{array}{c} \frac{1}{B^{2}}=\frac{1}{{\left(\frac{\sigma_{1}}{\sqrt{\gamma }}\right)}^{2}}+\frac{1}{\sigma_{2}^{2}}\\ \frac{A}{B^{2}}=\left(\frac{\mu_{1}}{{\left(\frac{\sigma_{1}}{\sqrt{\gamma }}\right)}^{2}}+\frac{\mu_{2}}{\sigma_{2}^{2}}\right) \end{array}\right..$$

Then,

$$A\left(\frac{1}{{\left(\frac{\sigma_{1}}{\sqrt{\gamma }}\right)}^{2}}+\frac{1}{\sigma_{2}^{2}}\right)=\frac{\mu_{1}}{{\left(\frac{\sigma_{1}}{\sqrt{\gamma }}\right)}^{2}}+\frac{\mu_{2}}{\sigma_{2}^{2}}$$

and

$$A=\frac{\frac{\mu_{1}}{{\left(\frac{\sigma_{1}}{\sqrt{\gamma }}\right)}^{2}}+\frac{\mu_{2}}{\sigma_{2}^{2}}}{\frac{1}{{\left(\frac{\sigma_{1}}{\sqrt{\gamma }}\right)}^{2}}+\frac{1}{\sigma_{2}^{2}}}=\frac{\frac{\sigma_{2}^{2}\mu_{1}+\mu_{2}{\left(\frac{\sigma_{1}}{\sqrt{\gamma }}\right)}^{2}}{\sigma_{2}^{2}{\left(\frac{\sigma_{1}}{\sqrt{\gamma }}\right)}^{2}}}{\frac{\sigma_{2}^{2}+{\left(\frac{\sigma_{1}}{\sqrt{\gamma }}\right)}^{2}}{\sigma_{2}^{2}{\left(\frac{\sigma_{1}}{\sqrt{\gamma }}\right)}^{2}}}=\frac{\sigma_{2}^{2}\mu_{1}+\mu_{2}\frac{\sigma_{1}^{2}}{\gamma }}{\sigma_{2}^{2}+\frac{\sigma_{1}^{2}}{\gamma }}=\frac{\gamma \sigma_{2}^{2}\mu_{1}+\mu_{2}\sigma_{1}^{2}}{\gamma \sigma_{2}^{2}+\sigma_{1}^{2}}.$$

We have,

$$\frac{1}{B^{2}}=\frac{1}{{\left(\frac{\sigma_{1}}{\sqrt{\gamma }}\right)}^{2}}+\frac{1}{\sigma_{2}^{2}}=\frac{\gamma }{\sigma_{1}^{2}}+\frac{1}{\sigma_{2}^{2}}=\frac{\sigma_{2}^{2}\gamma +\sigma_{1}^{2}}{\sigma_{1}^{2}\sigma_{2}^{2}}$$

Therefore,

$$B=\frac{\sigma_{1}\sigma_{2}}{\sqrt{\sigma_{2}^{2}\gamma +\sigma_{1}^{2}}}.$$

On the other hand,

$$\frac{A^{2}}{B^{2}}={\left(\frac{\gamma \sigma_{2}^{2}\mu_{1}+\mu_{2}\sigma_{1}^{2}}{\gamma \sigma_{2}^{2}+\sigma_{1}^{2}}\right)}^{2}\frac{\sigma_{2}^{2}\gamma +\sigma_{1}^{2}}{\sigma_{1}^{2}\sigma_{2}^{2}}=\frac{{\left(\gamma \sigma_{2}^{2}\mu_{1}+\mu_{2}\sigma_{1}^{2}\right)}^{2}}{\left(\gamma \sigma_{2}^{2}+\sigma_{1}^{2}\right)\sigma_{1}^{2}\sigma_{2}^{2}}$$

and

$$\begin{array}{ccc} L_{3} & = & \frac{1}{\sigma_{1}^{\gamma }{\left(\sqrt{2\pi }\right)}^{\gamma }}\frac{1}{\sigma_{2}\sqrt{2\pi }}\exp\left\{-\frac{1}{2}\left[\frac{\mu_{1}^{2}}{{\left(\frac{\sigma_{1}}{\sqrt{\gamma }}\right)}^{2}}+\frac{\mu_{2}^{2}}{\sigma_{2}^{2}}\right]\right\}\exp\left\{\frac{1}{2}\frac{A^{2}}{B^{2}}\right\}B\sqrt{2\pi }\\ & = & \frac{1}{\sigma_{1}^{\gamma }{\left(\sqrt{2\pi }\right)}^{\gamma }}\frac{1}{\sigma_{2}}\exp\left\{-\frac{1}{2}\left[\frac{\mu_{1}^{2}}{{\left(\frac{\sigma_{1}}{\sqrt{\gamma }}\right)}^{2}}+\frac{\mu_{2}^{2}}{\sigma_{2}^{2}}\right]\right\}\exp\left\{\frac{1}{2}\frac{{\left(\gamma \sigma_{2}^{2}\mu_{1}+\mu_{2}\sigma_{1}^{2}\right)}^{2}}{\left(\gamma \sigma_{2}^{2}+\sigma_{1}^{2}\right)\sigma_{1}^{2}\sigma_{2}^{2}}\right\}\frac{\sigma_{1}\sigma_{2}}{\sqrt{\sigma_{2}^{2}\gamma +\sigma_{1}^{2}}}\\ & = & \frac{\sigma_{1}\sigma_{2}}{\sqrt{\sigma_{2}^{2}\gamma +\sigma_{1}^{2}}}\frac{1}{\sigma_{1}^{\gamma }{\left(\sqrt{2\pi }\right)}^{\gamma }}\frac{1}{\sigma_{2}}\exp\left\{\frac{1}{2}\left[\frac{{\left(\gamma \sigma_{2}^{2}\mu_{1}+\mu_{2}\sigma_{1}^{2}\right)}^{2}}{\left(\gamma \sigma_{2}^{2}+\sigma_{1}^{2}\right)\sigma_{1}^{2}\sigma_{2}^{2}}-\frac{\gamma \mu_{1}^{2}\sigma_{2}^{2}+\sigma_{1}^{2}\mu_{2}^{2}}{\sigma_{2}^{2}\sigma_{1}^{2}}\right]\right\}. \end{array}$$

However,

$$\begin{array}{ccc} \frac{{\left(\gamma \sigma_{2}^{2}\mu_{1}+\mu_{2}\sigma_{1}^{2}\right)}^{2}}{\left(\gamma \sigma_{2}^{2}+\sigma_{1}^{2}\right)\sigma_{1}^{2}\sigma_{2}^{2}}-\frac{\gamma \mu_{1}^{2}\sigma_{2}^{2}+\sigma_{1}^{2}\mu_{2}^{2}}{\sigma_{2}^{2}\sigma_{1}^{2}} & = & \frac{{\left(\gamma \sigma_{2}^{2}\mu_{1}+\mu_{2}\sigma_{1}^{2}\right)}^{2}-\left(\gamma \mu_{1}^{2}\sigma_{2}^{2}+\sigma_{1}^{2}\mu_{2}^{2}\right)\left(\gamma \sigma_{2}^{2}+\sigma_{1}^{2}\right)}{\left(\gamma \sigma_{2}^{2}+\sigma_{1}^{2}\right)\sigma_{1}^{2}\sigma_{2}^{2}}\\ & = & \frac{\gamma^{2}\sigma_{2}^{4}\mu_{1}^{2}+\mu_{2}^{2}\sigma_{1}^{4}+2\gamma \sigma_{2}^{2}\mu_{1}\mu_{2}\sigma_{1}^{2}}{\left(\gamma \sigma_{2}^{2}+\sigma_{1}^{2}\right)\sigma_{1}^{2}\sigma_{2}^{2}}\\ & & -\frac{\gamma^{2}\mu_{1}^{2}\sigma_{2}^{4}+\gamma \mu_{1}^{2}\sigma_{2}^{2}\sigma_{1}^{2}+\mu_{2}^{2}\gamma \sigma_{2}^{2}\sigma_{1}^{2}+\mu_{2}^{2}\sigma_{1}^{4}}{\left(\gamma \sigma_{2}^{2}+\sigma_{1}^{2}\right)\sigma_{1}^{2}\sigma_{2}^{2}}\\ & = & \frac{2\gamma \sigma_{2}^{2}\mu_{1}\mu_{2}\sigma_{1}^{2}-\gamma \mu_{1}^{2}\sigma_{2}^{2}\sigma_{1}^{2}-\mu_{2}^{2}\gamma \sigma_{2}^{2}\sigma_{1}^{2}}{\left(\gamma \sigma_{2}^{2}+\sigma_{1}^{2}\right)\sigma_{1}^{2}\sigma_{2}^{2}}\\ & = & \frac{\sigma_{2}^{2}\sigma_{1}^{2}\gamma \left(2\mu_{1}\mu_{2}-\mu_{1}^{2}-\mu_{2}^{2}\right)}{\left(\gamma \sigma_{2}^{2}+\sigma_{1}^{2}\right)\sigma_{1}^{2}\sigma_{2}^{2}}\\ & = & -\frac{\gamma {\left(\mu_{1}-\mu_{2}\right)}^{2}}{\left(\gamma \sigma_{2}^{2}+\sigma_{1}^{2}\right)} \end{array}$$

Therefore,

$$L_{3}=\frac{1}{\sigma_{1}^{\gamma -1}\sqrt{\sigma_{2}^{2}\gamma +\sigma_{1}^{2}}}\frac{1}{{\left(\sqrt{2\pi }\right)}^{\gamma }}\exp\left\{-\frac{1}{2}\frac{\gamma {\left(\mu_{1}-\mu_{2}\right)}^{2}}{\left(\gamma \sigma_{2}^{2}+\sigma_{1}^{2}\right)}\right\}.$$

Then,

$$\begin{array}{ccc} R_{\gamma }\left(N\left(\mu_{1},\sigma_{1}\right),N\left(\mu_{2},\sigma_{2}\right)\right) & = & \frac{1}{\gamma +1}\ln L_{1}+\frac{1}{\gamma \left(\gamma +1\right)}\ln L_{2}-\frac{1}{\gamma }\ln L_{3}\\ & = & \frac{1}{\gamma +1}\ln\frac{1}{\sigma_{1}^{\gamma }{\left(\sqrt{2\pi }\right)}^{\gamma }}\frac{1}{\sqrt{\gamma +1}}+\frac{1}{\gamma \left(\gamma +1\right)}\ln\frac{1}{\sigma_{2}^{\gamma }{\left(\sqrt{2\pi }\right)}^{\gamma }}\frac{1}{\sqrt{\gamma +1}}\\ & & -\frac{1}{\gamma }\ln\frac{1}{\sigma_{1}^{\gamma -1}\sqrt{\sigma_{2}^{2}\gamma +\sigma_{1}^{2}}}\frac{1}{{\left(\sqrt{2\pi }\right)}^{\gamma }}+\frac{1}{2}\frac{\gamma {\left(\mu_{1}-\mu_{2}\right)}^{2}}{\gamma \left(\gamma \sigma_{2}^{2}+\sigma_{1}^{2}\right)}\\ & = & \frac{1}{\gamma \left(\gamma +1\right)}\left(\ln\frac{\sigma_{1}^{\gamma -1}}{\sigma_{2}^{\gamma }\sqrt{\gamma +1}}\sqrt{\sigma_{1}^{2}+\gamma \sigma_{2}^{2}}+\gamma \ln\frac{1}{\sigma_{1}\sqrt{\gamma +1}}\sqrt{\sigma_{1}^{2}+\gamma \sigma_{2}^{2}}\right)\\ & & +\frac{1}{2}\frac{\gamma {\left(\mu_{1}-\mu_{2}\right)}^{2}}{\gamma \left(\gamma \sigma_{2}^{2}+\sigma_{1}^{2}\right)}\\ & = & \frac{1}{\gamma \left(\gamma +1\right)}\ln\frac{1}{\sigma_{1}\sigma_{2}^{\gamma }}{\left(\frac{\sqrt{\sigma_{1}^{2}+\gamma \sigma_{2}^{2}}}{\sqrt{\gamma +1}}\right)}^{\gamma +1}+\frac{1}{2}\frac{{\left(\mu_{1}-\mu_{2}\right)}^{2}}{\left(\gamma \sigma_{2}^{2}+\sigma_{1}^{2}\right)} \end{array}$$

For γ→0 we have,

(A11) $$\lim_{\gamma \to 0}R_{\gamma }\left(N\left(\mu_{1},\sigma_{1}\right),N\left(\mu_{2},\sigma_{2}\right)\right)=\frac{\sigma_{2}^{2}-\sigma_{1}^{2}}{2\sigma_{1}^{2}}+\ln\frac{\sigma_{1}}{\sigma_{2}}+\frac{1}{2}\frac{{\left(\mu_{1}-\mu_{2}\right)}^{2}}{\sigma_{1}^{2}}.$$

### Appendix A.4. Computation of the Nonzero Eigenvalues of **A**_γ_*(***θ**_*0*_*)***B**_τ_*(***θ**_*0*_*)***K**_τ_*(***θ**_*0*_*)***B**_τ_*(***θ**_*0*_*)*

We know that the matrix ξ(θ) can be expressed as

$$\xi \left(\theta \right)=c_{\tau }\left(\theta \right)\kappa \left(\theta \right)$$

with

$$\kappa \left(\theta \right)=\int f_{\theta }{\left(x\right)}^{\tau +1}dx=\frac{1}{\sigma^{\tau }{\left(\sqrt{2\pi }\right)}^{\tau }\sqrt{1+\tau }}.$$

Then,

$$\xi \left(\theta \right)=\frac{1}{\sigma^{\tau }{\left(\sqrt{2\pi }\right)}^{\tau }\sqrt{1+\tau }}{\left(0,-\frac{\tau }{\left(\tau +1\right)}\frac{1}{\sigma }\right)}^{T}.$$

Therefore,

$$c_{\tau }\left(\theta \right)=\frac{\xi (\theta )}{\kappa (\theta )}=\left(0,-\frac{\tau }{\left(\tau +1\right)}\frac{1}{\sigma }\right).$$

On the other hand

$$\frac{\partial \log f_{\mu ,\sigma }\left(X_{i}\right)}{\partial \mu }=\frac{X_{i}-\mu }{\sigma^{2}}\;\mathrm{and}\;\frac{\partial \log f_{\mu ,\sigma }\left(X_{i}\right)}{\partial \sigma }=-\frac{1}{\sigma }+\frac{1}{\sigma^{3}}{\left(X_{i}-\mu \right)}^{2}$$

and

$$u_{\theta }\left(X_{i}\right)=\left(\frac{X_{i}-\mu }{\sigma^{2}},-\frac{1}{\sigma }+\frac{1}{\sigma^{3}}{\left(X_{i}-\mu \right)}^{2}\right).$$

Then,

$$\Psi_{\tau }\left(X;\theta \right)=\left(\Psi_{\tau }^{1}\left(X;\theta \right),\Psi_{\tau }^{2}\left(X;\theta \right)\right)$$

is given by

$$\Psi_{\tau }\left(X;\theta \right)=\left(\frac{X-\mu }{\sigma^{2}}\frac{1}{{\left(\sigma \sqrt{2\pi }\right)}^{\tau }}e^{-\frac{\tau }{2}{\left(\frac{X-\mu }{\sigma }\right)}^{2}},\left({\left(\frac{X-\mu }{\sigma }\right)}^{2}-\frac{1}{1+\tau }\right)\frac{1}{\sigma }\frac{1}{{\left(\sigma \sqrt{2\pi }\right)}^{\tau }}e^{-\frac{\tau }{2}{\left(\frac{X-\mu }{\sigma }\right)}^{2}}\right)$$

and

$$K_{\tau }\left(\theta \right)=E\left[\Psi_{\tau }\left(X;\theta \right)\Psi_{\tau }{\left(X;\theta \right)}^{T}\right].$$

Now we obtain the elements of that matrix,

$$\begin{array}{ccc} K_{\tau }^{11}\left(\theta \right) & = & E\left[{\left(\frac{X-\mu }{\sigma^{2}}\right)}^{2}\frac{1}{{\left(\sigma \sqrt{2\pi }\right)}^{2\tau }}e^{-\frac{2\tau }{2}{\left(\frac{X-\mu }{\sigma }\right)}^{2}}\right]\\ & = & \frac{1}{{\left(\sigma \sqrt{2\pi }\right)}^{2\tau }{\left(1+2\tau \right)}^{3/2}}\frac{1}{\sigma^{2}} \end{array}$$

$$\begin{array}{ccc} K_{\tau }^{12}\left(\theta \right) & = & K_{\tau }^{21}\left(\theta \right)=E\left[\left(\frac{X-\mu }{\sigma^{2}}\right)\left({\left(\frac{X-\mu }{\sigma }\right)}^{2}-\frac{1}{1+\tau }\right)\frac{1}{\sigma }\frac{1}{{\left(\sigma \sqrt{2\pi }\right)}^{2\tau }}e^{-\frac{2\tau }{2}{\left(\frac{X-\mu }{\sigma }\right)}^{2}}\right]\\ & = & 0 \end{array}$$

and

$$\begin{array}{ccc} K_{\tau }^{22}\left(\theta \right) & = & E\left[{\left({\left(\frac{X-\mu }{\sigma }\right)}^{2}-\frac{1}{1+\tau }\right)}^{2}\frac{1}{\sigma^{2}}\frac{1}{{\left(\sigma \sqrt{2\pi }\right)}^{2\tau }}e^{-\frac{2\tau }{2}{\left(\frac{X-\mu }{\sigma }\right)}^{2}}\right]\\ & = & \frac{1}{\sigma^{2}}\frac{3\tau^{2}+2+4\tau }{{\left(\sigma \sqrt{2\pi }\right)}^{2\tau }{\left(1+2\tau \right)}^{5/2}{\left(1+\tau \right)}^{2}} \end{array}$$

and

$$\begin{array}{ccc} K_{\tau }\left(\theta \right) & = & \left(\begin{array}{cc} \frac{1}{{\left(\sigma \sqrt{2\pi }\right)}^{2\tau }{\left(1+2\tau \right)}^{3/2}}\frac{1}{\sigma^{2}} & 0\\ 0 & \frac{1}{\sigma^{2}}\frac{3\tau^{2}+2+4\tau }{{\left(\sigma \sqrt{2\pi }\right)}^{2\tau }{\left(1+2\tau \right)}^{5/2}{\left(1+\tau \right)}^{2}} \end{array}\right)\\ & = & \frac{1}{\sigma^{2}}\frac{1}{{\left(\sigma \sqrt{2\pi }\right)}^{2\tau }{\left(1+2\tau \right)}^{3/2}}\left(\begin{array}{cc} 1 & 0\\ 0 & \frac{3\tau^{2}+2+4\tau }{{\left(1+\tau \right)}^{2}\left(1+2\tau \right)} \end{array}\right). \end{array}$$

Now we obtain the matrix $S_{\tau }\left(\theta \right).$ We have

$$\xi \left(\theta \right)=c_{\tau }\left(\theta \right)\kappa \left(\theta \right)$$

with

$$\kappa \left(\theta \right)=\int f_{\theta }{\left(x\right)}^{\tau +1}dx=\frac{1}{\sigma^{\tau }{\left(\sqrt{2\pi }\right)}^{\tau }\sqrt{1+\tau }}.$$

Then,

$$\xi \left(\theta \right)=\frac{1}{\sigma^{\tau }{\left(\sqrt{2\pi }\right)}^{\tau }\sqrt{1+\tau }}{\left(0,-\frac{\tau }{\left(\tau +1\right)}\frac{1}{\sigma }\right)}^{T}$$

and

$$\frac{1}{\kappa (\theta )}\xi \left(\theta \right)\xi {\left(\theta \right)}^{T}=\frac{1}{\sigma^{\tau +2}{\left(\sqrt{2\pi }\right)}^{\tau }\sqrt{1+\tau }}\left(\begin{array}{cc} 0 & 0\\ 0 & \frac{\tau^{2}}{{\left(\tau +1\right)}^{2}} \end{array}\right).$$

On the other hand

$$J_{\tau }\left(\theta \right)=E\left[\left(\begin{array}{cc} \frac{1}{\sigma^{4}}{\left(X-\mu \right)}^{2} & \frac{1}{\sigma^{2}}\left(\frac{1}{\sigma }-\frac{1}{\sigma^{3}}{\left(X-\mu \right)}^{2}\right)\left(X-\mu \right)\\ \frac{1}{\sigma^{2}}\left(\frac{1}{\sigma }-\frac{1}{\sigma^{3}}{\left(X-\mu \right)}^{2}\right)\left(X-\mu \right) & {\left(\frac{1}{\sigma }-\frac{1}{\sigma^{3}}{\left(X-\mu \right)}^{2}\right)}^{2} \end{array}\right)\frac{1}{{\left(\sigma \sqrt{2\pi }\right)}^{\tau }}e^{-\frac{\tau }{2}{\left(\frac{X-\mu }{\sigma }\right)}^{2}}\right]$$

$$\begin{array}{ccc} J_{\tau }^{11}\left(\theta \right) & = & E\left[\frac{1}{\sigma^{4}}{\left(\mu -X\right)}^{2}\frac{1}{{\left(\sigma \sqrt{2\pi }\right)}^{\tau }}e^{-\frac{\tau }{2}{\left(\frac{X-\mu }{\sigma }\right)}^{2}}\right]=\frac{1}{\sigma^{\tau +2}}\frac{1}{{\left(\tau +1\right)}^{3/2}}\frac{1}{{\left(\sqrt{2\pi }\right)}^{\tau }}\\ J_{\tau }^{12}\left(\theta \right) & = & J_{\tau }^{21}\left(\theta \right)=0\\ J_{\tau }^{22}\left(\theta \right) & = & E\left[{\left(\frac{1}{\sigma }-\frac{1}{\sigma^{3}}{\left(\mu -X\right)}^{2}\right)}^{2}\frac{1}{{\left(\sigma \sqrt{2\pi }\right)}^{\tau }}e^{-\frac{\tau }{2}{\left(\frac{X-\mu }{\sigma }\right)}^{2}}\right]=\frac{1}{\sigma^{\tau +2}}\frac{1}{{\left(\sqrt{2\pi }\right)}^{\tau }}\frac{1}{\sqrt{1+\tau }}\frac{2+\tau^{2}}{{\left(1+\tau \right)}^{2}} \end{array}$$

Therefore

$$J_{\tau }\left(\theta \right)=\frac{1}{\sigma^{\tau +2}}\frac{1}{{\left(\sqrt{2\pi }\right)}^{\tau }}\frac{1}{\sqrt{1+\tau }}\left(\begin{array}{cc} \frac{1}{1+\tau } & 0\\ 0 & \frac{2+\tau^{2}}{{\left(1+\tau \right)}^{2}} \end{array}\right)$$

$$\begin{array}{cc} S_{\tau }\left(\theta \right) & =J_{\tau }\left(\theta \right)-\frac{1}{\kappa (\theta )}\xi \left(\theta \right)\xi {\left(\theta \right)}^{T}\\ & =\frac{1}{\sigma^{\tau +2}}\frac{1}{{\left(\sqrt{2\pi }\right)}^{\tau }}\frac{1}{\sqrt{1+\tau }}\left(\left(\begin{array}{cc} \frac{1}{1+\tau } & 0\\ 0 & \frac{2+\tau^{2}}{{\left(1+\tau \right)}^{2}} \end{array}\right)-\left(\begin{array}{cc} 0 & 0\\ 0 & \frac{\tau^{2}}{{\left(\tau +1\right)}^{2}} \end{array}\right)\right)\\ & =\frac{1}{\sigma^{\tau +2}}\frac{1}{{\left(\sqrt{2\pi }\right)}^{\tau }}\frac{1}{\sqrt{1+\tau }}\left(\begin{array}{cc} \frac{1}{1+\tau } & 0\\ 0 & \frac{2}{{\left(\tau +1\right)}^{2}} \end{array}\right) \end{array}$$

Now we have,

- The matrix ${\left[G{\left(\theta_{0}\right)}^{T}S_{\tau }{\left(\theta_{0}\right)}^{-1}G\left(\theta_{0}\right)\right]}^{-1}$$\left(G\left(\theta \right)={\left(0,1\right)}^{T}\right)$
  $$\begin{array}{ccc} G{\left(\theta_{0}\right)}^{T}S_{\tau }{\left(\theta_{0}\right)}^{-1}G\left(\theta_{0}\right) & = & \left(\begin{array}{cc} 0 & 1 \end{array}\right){\left(\frac{1}{\sigma^{\tau +2}}\frac{1}{{\left(\sqrt{2\pi }\right)}^{\tau }}\frac{1}{\sqrt{1+\tau }}\left(\begin{array}{cc} \frac{1}{1+\tau } & 0\\ 0 & \frac{2}{{\left(\tau +1\right)}^{2}} \end{array}\right)\right)}^{-1}\left(\begin{array}{c} 0\\ 1 \end{array}\right)\\ & = & \frac{1}{2}\sigma^{2}\sigma^{\tau }{\left(\tau +1\right)}^{\frac{5}{2}}{\left(\sqrt{2}\sqrt{\pi }\right)}^{\tau } \end{array}$$
- The matrix $Q_{\tau }\left(\theta_{0}\right)=S_{\tau }^{-1}\left(\theta_{0}\right)G\left(\theta_{0}\right){\left[G^{T}\left(\theta_{0}\right)S_{\tau }^{-1}\left(\theta_{0}\right)G\left(\theta_{0}\right)\right]}^{-1}$
  $$\begin{array}{ccc} Q_{\tau }\left(\theta_{0}\right) & = & {\left(\frac{1}{\sigma^{\tau +2}}\frac{1}{{\left(\sqrt{2\pi }\right)}^{\tau }}\frac{1}{\sqrt{1+\tau }}\left(\begin{array}{cc} \frac{1}{1+\tau } & 0\\ 0 & \frac{2}{{\left(\tau +1\right)}^{2}} \end{array}\right)\right)}^{-1}\left(\begin{array}{c} 0\\ 1 \end{array}\right){\left(\frac{1}{2}\sigma^{2}\sigma^{\tau }{\left(\tau +1\right)}^{\frac{5}{2}}{\left(\sqrt{2}\sqrt{\pi }\right)}^{\tau }\right)}^{-1}\\ & = & \left(\begin{array}{c} 0\\ 1 \end{array}\right) \end{array}$$
- The matrix $B_{\tau }\left(\theta_{0}\right)=S_{\tau }{\left(\theta_{0}\right)}^{-1}G\left(\theta_{0}\right){\left[G{\left(\theta_{0}\right)}^{T}S_{\tau }{\left(\theta_{0}\right)}^{-1}G\left(\theta_{0}\right)\right]}^{-1}G{\left(\theta_{0}\right)}^{T}S_{\tau }{\left(\theta_{0}\right)}^{-1}=Q_{\tau }\left(\theta_{0}\right)G{\left(\theta_{0}\right)}^{T}S_{\tau }{\left(\theta_{0}\right)}^{-1}$
  $$\begin{array}{ccc} B_{\tau }\left(\theta_{0}\right) & = & Q_{\tau }\left(\theta_{0}\right)G^{T}\left(\theta_{0}\right)S_{\tau }^{-1}\left(\theta_{0}\right)=\left(\begin{array}{c} 0\\ 1 \end{array}\right)\left(\begin{array}{cc} 0 & 1 \end{array}\right){\left(\frac{1}{\sigma^{\tau +2}}\frac{1}{{\left(\sqrt{2\pi }\right)}^{\tau }}\frac{1}{\sqrt{1+\tau }}\left(\begin{array}{cc} \frac{1}{1+\tau } & 0\\ 0 & \frac{2}{{\left(\tau +1\right)}^{2}} \end{array}\right)\right)}^{-1}\\ & = & \left(\begin{array}{cc} 0 & 0\\ 0 & \frac{1}{2}\sigma^{2}\sigma^{\tau }{\left(\tau +1\right)}^{\frac{5}{2}}{\left(\sqrt{2}\sqrt{\pi }\right)}^{\tau } \end{array}\right) \end{array}$$
- The matrix $M_{\gamma ,\tau }\left(\theta_{0}\right)=\frac{S_{\gamma }\left(\theta_{0}\right)}{\kappa_{\gamma }\left(\theta_{0}\right)}B_{\tau }\left(\theta_{0}\right)K_{\tau }\left(\theta_{0}\right)B_{\tau }\left(\theta_{0}\right)$
  $$\begin{array}{ccc} M_{\gamma ,\tau }\left(\theta_{0}\right) & = & \frac{\sigma^{\gamma }{\left(\sqrt{2\pi }\right)}^{\gamma }\sqrt{1+\gamma }}{\sigma^{\gamma +2}}\frac{1}{{\left(\sqrt{2\pi }\right)}^{\gamma }}\frac{1}{\sqrt{1+\gamma }}\left(\begin{array}{cc} \frac{1}{1+\gamma } & 0\\ 0 & \frac{2}{{\left(\gamma +1\right)}^{2}} \end{array}\right)\\ & & \times \left(\begin{array}{cc} 0 & 0\\ 0 & \frac{1}{2}\sigma^{2}\sigma^{\tau }{\left(\tau +1\right)}^{\frac{5}{2}}{\left(\sqrt{2}\sqrt{\pi }\right)}^{\tau } \end{array}\right)\\ & & \times \frac{1}{\sigma^{2}}\frac{1}{{\left(\sigma \sqrt{2\pi }\right)}^{2\tau }{\left(1+2\tau \right)}^{3/2}}\left(\begin{array}{cc} 1 & 0\\ 0 & \frac{3\tau^{2}+2+4\tau }{{\left(1+\tau \right)}^{2}\left(1+2\tau \right)} \end{array}\right)\\ & & \times \left(\begin{array}{cc} 0 & 0\\ 0 & \frac{1}{2}\sigma^{2}\sigma^{\tau }{\left(\tau +1\right)}^{\frac{5}{2}}{\left(\sqrt{2}\sqrt{\pi }\right)}^{\tau } \end{array}\right)\\ & = & \left(\begin{array}{cc} 0 & 0\\ 0 & \frac{1}{2}\frac{{\left(\tau +1\right)}^{3}}{{\left(\gamma +1\right)}^{2}{\left(2\tau +1\right)}^{\frac{5}{2}}}\left(3\tau^{2}+4\tau +2\right) \end{array}\right). \end{array}$$
